# Machine Learning-Based Screening for Obstructive Sleep Apnea Using Photographic Craniofacial and Speech-Derived Acoustic Features

**DOI:** 10.3390/life16101748

**Published:** 2026-10-18

**Authors:** Marissa López-Zaldivar, Sulema Torres-Ramos, Israel Román-Godínez, Daniel Hernández-Gordillo, Aurora Sofia Vázquez-Jiménez, Ricardo Antonio Salido-Ruiz

**Affiliations:** 1 Universidad de Guadalajara, Centro Universitario de Ciencias Exactas e Ingenierías, Departamento de Bioingeniería Traslacional, Guadalajara 44430, Jalisco, Mexico; marissa.lopez7953@alumnos.udg.mx; 2 Universidad de Guadalajara, Centro Universitario de Ciencias Exactas e Ingenierías, Departamento de Ciencias Computacionales, Guadalajara 44430, Jalisco, Mexico; sulema.torres@academicos.udg.mx; 3 Universidad de Guadalajara, Centro Universitario de Ciencias Exactas e Ingenierías, Departamento de Innovación Basada en la Información y el Conocimiento, Guadalajara 44430, Jalisco, Mexico; israel.roman@academicos.udg.mx; 4 UMAE Hospital de Especialidades, Centro Médico Nacional de Occidente, IMSS, Guadalajara 44340, Jalisco, Mexico; danielhdzgo@gmail.com (D.H.-G.); auro_sofy_612@hotmail.com (A.S.V.-J.)

**Keywords:** obstructive sleep apnea, craniofacial descriptors, acoustic features, speech analysis, machine learning, screening, apnea–hypopnea index

## Abstract

Obstructive sleep apnea (OSA) is a prevalent disorder for which accessible non-invasive screening approaches remain of interest. This study evaluated photographic craniofacial descriptors and speech-derived acoustic features for Apnea–Hypopnea Index (AHI) estimation and binary OSA screening. Data from 109 adults were analyzed using unsegmented and temporally segmented speech representations. Seven craniofacial, acoustic, and combined feature configurations were evaluated using nested cross-validation. For regression, the lowest MAE was 14.242 events/h using segmented continuous-speech features with Random Forest. For classification, the highest AUC-ROC was 0.766 using unsegmented craniofacial features with a multilayer perceptron classifier, while the best segmented result was 0.756 using craniofacial plus continuous-speech features. Craniofacial-only features in the segmented representation achieved a similar AUC-ROC of 0.753, indicating no clear discriminative advantage from adding acoustic features. Several classifiers showed high sensitivity but comparatively low specificity. Sensitivity analysis excluding the four participants with the highest AHI values showed similar classification patterns, although regression performance was more sensitive to cohort composition. Overall, craniofacial descriptors provided the most consistent predictive contribution, whereas acoustic features showed model- and representation-dependent value for OSA screening.

## 1. Introduction

Obstructive sleep apnea (OSA) is a common sleep-related breathing disorder associated with neurological, cardiovascular, and metabolic complications [1]. OSA represents the most prevalent sleep-related breathing disorder in adults [2]. In the United States, the reported prevalence is 17% among women and 34% among men, with similar rates observed in other countries [3]. According to a representative cross-sectional study published in 2018, one in four adults in Mexico is at high risk of OSA [4]. Beyond its high prevalence, OSA is strongly associated with metabolic and cardiovascular disorders, including metabolic syndrome, obesity, hypertension, insulin resistance, and dyslipidemia [5]. Studies have shown that the prevalence of metabolic syndrome among patients with OSA ranges from 50% to 80% across Asian, Caucasian, and Mediterranean populations [5].

Typically, OSA does not exhibit obvious physical signs during clinical examination; therefore, the initial diagnostic evaluation relies on screening questionnaires such as the Berlin Questionnaire, the STOP-BANG Questionnaire, and the NoSAS Questionnaire [2,3]. Patients often report excessive daytime sleepiness, fatigue, nocturnal gastroesophageal reflux, morning headaches, and snoring. However, a definitive diagnosis generally requires evaluation at a specialized sleep clinic [6]. Clinical assessment includes polysomnography (PSG) or respiratory polygraphy (RP), which record multiple physiological signals related primarily to respiration during sleep, including electroencephalography (EEG), electrocardiography (ECG), electrooculography (EOG), blood oxygen saturation (SpO_2_), submental electromyography (EMG), airflow, among others. The primary outcome of these examinations is the Apnea–Hypopnea Index (AHI), which quantifies OSA severity [2].

Although PSG remains the gold standard for OSA diagnosis, its cost, inconvenience, and limited accessibility have motivated the development of reliable non-invasive alternatives [4]. Recent advances in artificial intelligence have enabled machine learning models capable of extracting clinically relevant information from non-invasive biometric data, providing new opportunities to support clinical decision-making during OSA assessment. Among these approaches, machine learning and deep learning techniques have been applied to ECG, facial images, speech signals, clinical variables, and questionnaire responses [7,8,9,10,11,12].

Balaei et al. have provided evidence that OSA is related to craniofacial structure [13], while Perero-Codosero et al. have associated it with abnormalities in speech [14]. On the other hand, recent work by Pang et al. demonstrates that the characterization of high-frequency components in speech signals alone performs well for OSA identification [15]. Among multimodal approaches, Espinoza-Cuadros et al. compared the combination of clinical variables and speech signals versus the combination of clinical variables and local craniofacial features for assessing OSA severity [11]. A low-dimensional representation based on i-vectors was used to characterize the acoustic information. Subsequently, Support Vector Regression (SVR) was applied to the clinical variables and to the feature vectors extracted from acoustic and cephalometric characteristics independently to estimate the AHI. The study concluded that combining clinical variables with cephalometric characteristics improved OSA severity prediction, while the direct integration of craniofacial and acoustic biomarkers remained largely unexplored.

Together, these studies demonstrate that craniofacial characteristics and speech signals are individually informative for OSA prediction. However, the complementary value of integrating both non-invasive biometric modalities has been only sparsely investigated, particularly without relying on conventional clinical variables. In this context, this study evaluates the individual and combined contributions of craniofacial measurements and acoustic features to machine learning-based non-invasive OSA screening. To address this objective, supervised machine learning models were developed for both AHI regression and binary OSA classification. The proposed approach aims to evaluate the potential of non-invasive craniofacial and speech-derived information to support OSA screening alongside conventional diagnostic procedures. Such approaches may contribute to more accessible preliminary assessment and help identify individuals who could benefit from further clinical evaluation. Additionally, this study evaluates the proposed approach in a Mexican clinical population that includes both men and women, extending previous work that was restricted to Caucasian male participants [11].

## 2. Materials and Methods

The overall methodology consisted of four main stages, as illustrated in Figure 1. The first stage comprised the design of the acquisition protocol, approval by the institutional ethics committee, and collection of craniofacial photographs and speech recordings. The second stage involved data preprocessing, including speech signal filtering and segmentation, as well as automatic facial landmark detection followed by expert verification. The third stage focused on feature extraction from both data modalities and the construction of the experimental datasets. Finally, machine learning models were trained, optimized, and evaluated for both Apnea–Hypopnea Index (AHI) regression and binary OSA classification (AHI <10 vs. AHI ≥10). Model performance was subsequently compared across different craniofacial, acoustic, and multimodal feature configurations using quantitative performance metrics.

### 2.1. Data Acquisition

A standardized acquisition protocol was implemented after approval of the study by the IMSS Research Ethics Committee (approval number R-2023-785-082). The study was conducted at the Sleep Clinic of the Specialty Hospital, Western National Medical Center (Centro Médico Nacional de Occidente, CMNO), Mexican Social Security Institute (IMSS), Guadalajara, Jalisco, Mexico.

Frontal and lateral facial photographs, together with synchronized video recordings from which the speech signals were extracted, were acquired using two GoPro HERO10 Black cameras and a RØDE VideoMic GO microphone. All photographs and video recordings were acquired by medical doctors from the Pulmonology Division at the Specialty Hospital. The acquisition setup and overall workflow are illustrated in Figure 2 and Figure 3, respectively.

Based on previous work [11], a standardized acquisition protocol was designed to collect both craniofacial photographs and speech recordings from each participant. Two facial photographs were acquired for each subject, one frontal and one lateral. For speech acquisition, a set of linguistically balanced Spanish phrases previously proposed for identifying speech anomalies associated with OSA was adopted [16]. These phrases were selected because they include phonetic and articulatory contexts that may reveal abnormalities in articulation, phonation, and resonance associated with OSA. The protocol included the following four phrases in Spanish:

1. “Francia, Suiza y Hungría ya hicieron causa común”;
2. “Julián no vio la manga roja que ellos buscan, en ningún almacén”;
3. “Juan no puso la taza rota que tanto le gusta en el aljíbe”;
4. “Miguel y Manu llamarán entre ocho y nueve y media”.

To complement the continuous-speech recordings, participants were also instructed to produce sustained phonations of the five Spanish vowels, maintaining each vowel for at least three seconds. The acquisition procedure was guided by an instructional video that standardized both the recording sequence and timing. A 2500 Hz beep was emitted immediately before each utterance to facilitate the subsequent segmentation of the nine speech segments of interest (four continuous-speech phrases and five sustained vowels).

### 2.2. Data Preprocessing

Because the two data modalities required different processing strategies, preprocessing was performed independently for speech signals and facial images.

#### 2.2.1. Speech Signals Preprocessing

Figure 4 illustrates a sample speech signal obtained from the data.

The recorded speech signals contained synchronization beeps that were used to identify the beginning of each speech segment. Therefore, a band-pass filter with lower and upper cutoff frequencies of 2480 Hz and 2520 Hz, respectively, was applied to automatically detect these synchronization beeps. Figure 5 illustrates the detected synchronization beeps after band-pass filtering.

The Hilbert transform was used to obtain the signal envelope [17], enabling the identification of vocalization intervals and the removal of non-speech segments. Using the detected synchronization beeps as temporal references, each recording was segmented into the nine speech segments of interest. Figure 6 illustrates the segmentation points used to isolate these segments.

Finally, zero-crossing points were identified for each segment to remove portions of the signal containing silence. For each participant, the resulting speech segments were stored in a folder identified by a unique study identifier. Each folder contained nine audio files: five sustained vowels and four continuous-speech phrases.

#### 2.2.2. Facial Image Preprocessing

Using the *iOSA* (Version 1.0) software, developed in collaboration with Computer Engineering students at the University of Guadalajara, the facial reference points described in Table 1 were identified in the frontal and lateral photographs. For frontal images, landmark positions were initialized using MediaPipe Face Mesh; for lateral images, seven predefined landmark positions were displayed. Medical specialists from CMNO reviewed these positions and manually refined them through the *iOSA* interface when necessary. Whether landmark reviewers were blinded to the sleep-study results was not formally documented. Final landmark coordinates were stored as image-width- and image-height-normalized *x* and *y* coordinates. The schematic locations of the facial reference points in the frontal and lateral views are illustrated in Figure 7. Figure 8 shows the graphical interface used for landmark visualization and adjustment.

### 2.3. Feature Extraction

Following preprocessing, numerical descriptors were extracted from the finalized facial landmarks and the isolated speech segments.

#### 2.3.1. Craniofacial Measurements

The 13 craniofacial descriptors used in the machine-learning analyses were calculated from the frontal and lateral facial landmarks obtained with the *iOSA* software described in Section 2.2.2. The reference points used for these calculations are listed in Table 1, and the derived descriptors are summarized in Table 2.

Physical scaling was estimated using QR markers with a nominal size of 4.5 cm. For each detected marker, the width and height of its bounding rectangle were obtained. The mean of these dimensions across the detected markers was divided by 4.5 cm to derive a pixel-per-centimeter scale. When no QR marker was detected, the software used a fixed scale of 20.93 pixels/cm.

Linear measurements were calculated from horizontal differences between landmark *x* coordinates and converted to centimeters using the corresponding image width and pixel-per-centimeter scale. Thus, these descriptors represent horizontal projected distances rather than Euclidean distances. The frontal triangular-area descriptor was calculated as the two-dimensional area of the triangle defined by tr, tl, and snf after conversion of image coordinates to centimeters. Angular descriptors were calculated from two-dimensional landmark vectors using the dot product and arccosine and were expressed in degrees.

The variable internally named anb_angle was calculated from the vectors b→n and b→a and therefore corresponds geometrically to the n–b–a angle, with b as the vertex. It is therefore referred to here as the photographic n–b–a angle.

The software-derived variable originally labelled “middle cranial fossa volume” was calculated in two stages. First, the two-dimensional area of the lateral n–a–t triangle was calculated after conversion of the lateral-image coordinates to centimeters. This area was then multiplied by a width proxy derived from the normalized frontal tr–tl landmark coordinates. In the software implementation used to generate the study data, this width proxy was converted using the image width and pixel-per-centimeter scale associated with the lateral image, rather than the independently calculated frontal face-width measurement. The resulting quantity is expressed in cm^3^. Because this procedure does not involve direct three-dimensional segmentation or reconstruction of an internal cranial structure, the variable is referred to here as a photographic n–a–t area × frontal-landmark width proxy.

#### 2.3.2. Acoustic Features

The acoustic features extracted from each speech segment are summarized in Table 3. These features served as the acoustic input variables for the machine learning models.

Together, these craniofacial and acoustic descriptors constituted the input variables used to construct the experimental datasets described in the following subsection.

### 2.4. Experimental Datasets

Data were acquired from 109 participants, all of whom were included in the primary machine-learning analyses. Two experimental datasets were constructed from the same study population: an unsegmented dataset containing one instance per participant and a segmented dataset containing multiple temporally derived instances per participant. The construction of both datasets is described in the following subsections.

#### 2.4.1. Participant Characteristics

The demographic and clinical characteristics of the 109 participants are summarized in Table 4. Participants are presented for the complete cohort and stratified according to the binary classification threshold used in this study (AHI <10 vs. AHI ≥10 events/h). Age was calculated at the date of the sleep study, and body mass index (BMI) was calculated from measured weight and height.

The study population included 55 men and 54 women, with a mean age of 53.9±14.0 years and a mean BMI of 36.27±8.52 kg/m^2^. Descriptively, participants with AHI ≥10 events/h were older and had higher mean BMI and neck circumference than participants with AHI <10 events/h. Hypertension, snoring, and witnessed apneas were also more frequent in the AHI ≥10 group, and the mean Epworth Sleepiness Scale score was higher in this group.

A complete description of the demographic and clinical variables collected for the study population is provided in Supplementary Table S1. Missing clinical data are summarized in Supplementary Table S2. These clinical variables were collected to characterize the study population and were not included as predictors in the machine-learning models evaluated in this study.

#### 2.4.2. AHI Distribution and Binary Classification Groups

The distribution of participants according to AHI range and sex is presented in Table 5. Conventional AHI severity ranges were used to describe the clinical distribution of OSA in the study population, with the mild range further divided at 10 events/h to reflect the binary threshold used for the machine-learning classification task.

The threshold of 10 events/h was selected in consultation with the clinical team after data collection to define an operational binary screening task. This threshold was not selected through model-performance optimization and should not be interpreted as a replacement for conventional clinical OSA diagnostic or severity thresholds. An AHI threshold of 10 events/h has also been used in previous speech- and acoustic-based OSA classification studies [10].

Using this criterion, 34 participants (31.2%) were assigned to the AHI <10 group and 75 participants (68.8%) to the AHI ≥10 group. Consequently, the classification dataset presented a moderate class imbalance, which was explicitly considered during model training and performance evaluation, as described in Section 2.6.

#### 2.4.3. Unsegmented Dataset

For the unsegmented configuration, acoustic features were extracted from each complete speech segment without further temporal subdivision. Each participant contributed nine speech segments: five sustained vowels and four continuous-speech phrases. The 14 acoustic features described in Table 3 were extracted from each segment, resulting in 126 acoustic features per participant.

These acoustic descriptors were combined with the 13 craniofacial measurements described in Table 2, yielding a total of 139 input features per participant. Therefore, the unsegmented dataset contained one instance per participant and comprised 109 instances in the primary analysis.

#### 2.4.4. Segmented Dataset

To investigate whether temporal variations in speech could provide additional predictive information, a second dataset was generated by subdividing each of the nine speech segments into up to 30 overlapping frames with 50% overlap. The same acoustic features listed in Table 3 were extracted from every frame, whereas the craniofacial measurements remained unchanged for all frames belonging to the same participant.

For each frame index, the acoustic features extracted from the five sustained vowels and four continuous-speech phrases were concatenated to form a single segmented instance. Thus, each instance comprised 126 acoustic features (9 speech segments × 14 acoustic features) together with the same 13 craniofacial measurements used in the unsegmented configuration, yielding a total of 139 input features.

Participant identity was preserved for every segmented instance to ensure subject-level grouping during model training and evaluation, as described in Section 2.6. If all 109 participants had produced 30 valid segmented instances, the resulting dataset would have contained 3270 instances. However, some frames were too short to permit reliable acoustic feature extraction. When the features required to construct a given segmented instance could not be reliably obtained, that instance was not retained. Consequently, the final segmented dataset comprised 3153 valid instances.

A total of 117 segmented instances were discarded: 53 from participants in the AHI <10 group and 64 from participants in the AHI ≥10 group. The participant-level distribution of valid and discarded segmented instances is reported in Supplementary Table S3. During model evaluation, predictions from segmented instances were aggregated at the participant level before performance metrics were calculated, as described in Section 2.6.

### 2.5. Machine-Learning Models

To evaluate the predictive contribution of the different information sources, seven feature configurations were defined:

1. Craniofacial measurements (CF);
2. Sustained-vowel acoustic features (V);
3. Continuous-speech acoustic features (P);
4. Craniofacial measurements + sustained-vowel acoustic features (CF+V);
5. Craniofacial measurements + continuous-speech acoustic features (CF+P);
6. Sustained-vowel + continuous-speech acoustic features (V+P);
7. Craniofacial measurements + sustained-vowel + continuous-speech acoustic features (CF+V+P).

The same seven feature configurations were evaluated for both the unsegmented and segmented datasets. Two prediction tasks were considered: regression, in which the continuous AHI value was estimated, and binary classification, in which participants were assigned to the negative screening class (AHI <10 events/h) or the positive screening class (AHI ≥10 events/h).

For the classification task, nine machine-learning algorithms were considered: Support Vector Classifier (SVC), Gaussian Naive Bayes (GNB), Quadratic Discriminant Analysis (QDA), K-Nearest Neighbors (KNN), Decision Tree (DT), Random Forest (RF), Multi-layer Perceptron Classifier (MLPC), Logistic Regression Classifier (LRC), and Extreme Gradient Boosting Classifier (XGBC). QDA was evaluated only when the dimensionality of the feature representation permitted reliable estimation of class-specific covariance matrices.

For the regression task, eight algorithms were evaluated: Support Vector Regressor (SVR), Decision Tree (DT), Stochastic Gradient Descent Regressor (SGD), Lasso regression, Ridge regression, Random Forest Regressor (RF), Multi-layer Perceptron Regressor (MLPR), and Extreme Gradient Boosting Regressor (XGBR).

### 2.6. Model Training and Performance Evaluation

#### 2.6.1. Nested Cross-Validation and Hyperparameter Optimization

Model development and performance evaluation were conducted using nested cross-validation to reduce optimistic bias associated with hyperparameter tuning and model selection. The same evaluation framework was applied to both regression and classification tasks and to the unsegmented and segmented datasets.

The outer evaluation loop consisted of five folds and was repeated 10 times using different data partitions. Thus, each participant received an out-of-fold prediction once within each repeat. Hyperparameter optimization was performed exclusively within the training portion of each outer fold using a four-fold inner cross-validation procedure. Randomized Search Cross-Validation (RandomizedSearchCV) was used to evaluate up to 20 hyperparameter configurations for each algorithm from the predefined search spaces. The parameters included in the hyperparameter searches for each classification and regression algorithm are listed in Supplementary Table S4. Consequently, the outer test folds were not used for hyperparameter selection.

For the classification task, stratification was used to preserve the distribution of the AHI <10 and AHI ≥10 classes across folds. Class-imbalance handling was incorporated into the inner model-selection procedure. For classifiers supporting class weighting (SVC, DT, RF, and LRC), class_weight=None and class_weight=balanced were included as candidate hyperparameter settings. For XGBoost, scale_pos_weight was evaluated using either 1.0 or the class-imbalance ratio estimated from the corresponding training data. The imbalance-handling strategy was therefore selected exclusively from the training data within the inner cross-validation loop, without using information from the outer test fold.

For the segmented dataset, participant identity was used as a grouping variable in both the inner and outer cross-validation procedures. All segmented instances originating from a given participant were therefore assigned to the same fold. This prevented instances from the same participant from appearing simultaneously in training and test sets. Predictions generated for the segmented instances belonging to each participant were averaged to obtain a single participant-level prediction before performance metrics were calculated. Thus, both unsegmented and segmented configurations were evaluated at the participant level.

For each of the 10 outer cross-validation repeats, the out-of-fold predictions from the five outer test folds were concatenated to reconstruct a complete set of predictions for the study cohort. Performance metrics were then calculated from these complete out-of-fold predictions, yielding one participant-level estimate per metric for each repeat. The reported point estimate for each model and feature configuration was obtained by averaging the corresponding metric across the 10 repeats. Outer folds were therefore not treated as independent observations when estimating model performance.

#### 2.6.2. Performance Metrics, Baselines, and Confidence Intervals

For binary classification, the AHI ≥10 events/h group was considered the positive class and the AHI <10 events/h group the negative class. AUC-ROC was used as the primary classification metric and as the optimization criterion during inner cross-validation. Balanced accuracy, sensitivity, specificity, and accuracy were also reported to characterize classification performance.

Accuracy was computed as:

(1) $$\mathrm{Accuracy}=\frac{TP+TN}{TP+TN+FP+FN}$$

Sensitivity was computed as:

(2) $$\mathrm{Sensitivity}=\frac{TP}{TP+FN}$$

Specificity was computed as:

(3) $$\mathrm{Specificity}=\frac{TN}{TN+FP}$$

and balanced accuracy was computed as:

(4) $$\mathrm{Balanced}\;\mathrm{Accuracy}=\frac{\mathrm{Sensitivity}+\mathrm{Specificity}}{2}$$

where TP, TN, FP, and FN denote true positives, true negatives, false positives, and false negatives, respectively.

For regression, Mean Absolute Error (MAE) was defined as the primary performance metric and was also used as the optimization criterion during inner cross-validation. Root Mean Squared Error (RMSE) was calculated as a complementary performance measure.

MAE was computed as:

(5) $$\mathrm{MAE}=\frac{1}{n}\sum_{i=1}^{n}\left|y_{i}-{\hat{y}}_{i}\right|$$

where $y_{i}$ denotes the observed AHI value, ${\hat{y}}_{i}$ the predicted AHI value, and *n* the number of participants.

RMSE was computed as:

(6) $$\mathrm{RMSE}=\sqrt{\frac{1}{n}\sum_{i=1}^{n}{\left(y_{i}-{\hat{y}}_{i}\right)}^{2}}.$$

Uncertainty in model performance was quantified using nonparametric bootstrap confidence intervals at the participant level. For each model and feature configuration, 1000 bootstrap resamples of participants were generated with replacement. Within each bootstrap resample, the metric was recalculated from the corresponding complete out-of-fold predictions for each of the 10 repeats and then averaged across repeats. The 2.5th and 97.5th percentiles of the resulting bootstrap distribution were used to obtain the 95% confidence interval. Resampling was performed at the participant level rather than at the segmented-instance level to preserve participant independence.

For classification, performance was additionally compared with a majority-class baseline. For each outer fold, all test participants were assigned to the majority class observed in the corresponding outer-training fold. A constant non-discriminative score was used for the baseline AUC-ROC, corresponding to an expected AUC-ROC of 0.50. Because AHI ≥10 events/h was the majority class in the primary cohort, this baseline also provided a reference for interpreting accuracy and balanced accuracy.

For regression, a simple reference model was also evaluated. For each outer fold, the median AHI of the corresponding training participants was used as the prediction for all participants in the outer test fold. This baseline was evaluated using the same out-of-fold procedure as the machine-learning regression models.

Both baselines were evaluated separately for each cohort and representation, using the same outer cross-validation partitions as the corresponding machine-learning models. Baseline metrics were calculated from the complete participant-level out-of-fold predictions within each repeat and then averaged across the 10 repeats. Baseline confidence intervals were obtained using the participant-level bootstrap procedure described above.

#### 2.6.3. Sensitivity Analysis

A sensitivity analysis was conducted to assess whether the main findings depended on the inclusion of participants with the highest AHI values. The primary analysis included all 109 participants. For the sensitivity analysis, the four participants with AHI values of 104.3, 77.2, 75.9, and 74.7 events/h were excluded, yielding a cohort of 105 participants, comprising 71 participants in the positive screening class and 34 in the negative screening class according to the AHI threshold of 10 events/h.

To limit computational cost, the sensitivity analysis focused on six configurations selected from the primary analysis before evaluating the reduced cohort. These configurations were chosen to represent craniofacial-only, acoustic-only, and multimodal approaches, considering their performance in the primary analysis and coverage of both prediction tasks. The classification configurations were MLPC with unsegmented CF, MLPC with unsegmented V+P, and RF with segmented CF+P. The regression configurations were SVR with unsegmented CF, RF with segmented P, and SGD with segmented CF+P.

Each configuration was re-evaluated using the same preprocessing procedures, hyperparameter search spaces, random seed, and nested cross-validation framework described above. Hyperparameters were optimized within the training data of each outer fold. Participant grouping was maintained for segmented data, and performance was evaluated at the participant level.

Results from the 109- and 105-participant cohorts were compared descriptively using AUC-ROC for classification and MAE for regression, together with their 95% confidence intervals and the corresponding cohort-specific baselines. Comparisons focused on changes in these performance estimates and on performance relative to the baselines for the six selected configurations.

## 3. Results

### 3.1. Regression Results

#### 3.1.1. Unsegmented Dataset

Regression performance for the unsegmented dataset is summarized in Table 6. For each feature configuration, the model with the lowest mean MAE across the 10 complete outer cross-validation repeats is reported.

The lowest MAE was obtained using the craniofacial feature set (CF) with SVR, with an MAE of 14.537 events/h (95% CI: 12.239–17.059). Among the remaining feature configurations, CF+P achieved the next lowest MAE, with SGD reaching 15.327 events/h (95% CI: 12.956–17.848). The CF+V and CF+V+P configurations showed MAEs of 15.667 and 15.700 events/h, respectively.

Feature sets based exclusively on acoustic information showed higher errors: 16.328 events/h for V, 16.085 events/h for P, and 16.040 events/h for V+P. The median-AHI baseline yielded an MAE of 15.915 events/h (95% CI: 13.336–18.643).

Complete regression results for all evaluated algorithms and feature configurations for the unsegmented dataset are provided in Supplementary Table S5, while the corresponding RMSE results are reported in Supplementary Table S6.

#### 3.1.2. Segmented Dataset

Regression performance for the segmented dataset is summarized in Table 7. For each feature configuration, the model with the lowest mean MAE across the 10 complete outer cross-validation repeats is reported.

The lowest MAE was obtained using the continuous-speech feature set (P) with RF, with an MAE of 14.242 events/h (95% CI: 12.296–16.290). The CF configuration with SVR yielded an MAE of 14.713 events/h (95% CI: 12.349–17.072), while CF+P with SGD achieved an MAE of 14.761 events/h (95% CI: 12.336-17.615).

The remaining multimodal configurations showed MAEs of 15.150 events/h for CF+V, 15.116 events/h for CF+V+P, and 15.787 events/h for V+P. The V configuration yielded an MAE of 15.934 events/h. The median-AHI baseline produced an MAE of 15.871 events/h (95% CI: 13.257–18.673).

Complete regression results for all evaluated algorithms and feature configurations for the segmented dataset are provided in Supplementary Table S7, while the corresponding RMSE results are reported in Supplementary Table S8.

### 3.2. Classification Results

#### 3.2.1. Unsegmented Dataset

Classification performance for the unsegmented dataset is summarized in Table 8. For each feature configuration, the model with the highest mean AUC-ROC across the 10 complete outer cross-validation repeats is reported.

The highest AUC-ROC was obtained using the craniofacial feature set (CF) with MLPC, reaching 0.766 (95% CI: 0.671–0.846). Among the multimodal configurations, CF+V with RF achieved an AUC-ROC of 0.704 (95% CI: 0.599–0.802), followed by CF+P with RF at 0.685 (95% CI: 0.567–0.793) and CF+V+P with RF at 0.678 (95% CI: 0.569–0.784).

Feature sets based exclusively on acoustic information showed lower AUC-ROC values: 0.571 for V, 0.550 for P, and 0.578 for V+P. The majority-class baseline yielded an AUC-ROC of 0.500 and a balanced accuracy of 0.500.

AUC-ROC results for all evaluated algorithms and feature configurations are provided in Supplementary Table S9.

#### 3.2.2. Segmented Dataset

Classification performance for the segmented dataset is summarized in Table 9. For each feature configuration, the model with the highest mean AUC-ROC across the 10 complete outer cross-validation repeats is reported.

The highest AUC-ROC was obtained using the CF+P configuration with RF, reaching 0.756 (95% CI: 0.651–0.849). Similar AUC-ROC values were observed for CF with RF at 0.753 (95% CI: 0.655–0.841), CF+V with MLPC at 0.751 (95% CI: 0.651–0.838), and CF+V+P with LRC at 0.749 (95% CI: 0.652–0.836).

Feature sets based exclusively on acoustic information showed lower AUC-ROC values: 0.521 for V, 0.568 for P, and 0.510 for V+P. The majority-class baseline yielded an AUC-ROC of 0.500 and a balanced accuracy of 0.500.

AUC-ROC results for all evaluated algorithms and feature configurations are provided in Supplementary Table S10.

### 3.3. Sensitivity Analysis

The sensitivity analysis showed small changes in AUC-ROC across the three selected classification configurations (Table 10). AUC-ROC decreased from 0.766 to 0.750 for MLPC using unsegmented CF, from 0.578 to 0.577 for MLPC using unsegmented V+P, and from 0.756 to 0.755 for RF using segmented CF+P. In both cohorts, the selected MLPC–CF and RF–CF+P classification configurations had higher AUC-ROC point estimates than MLPC–V+P.

For regression, MAE decreased from 14.537 to 13.417 events/h for SVR using unsegmented CF and from 14.761 to 13.120 events/h for SGD using segmented CF+P, whereas RF using segmented P showed an almost unchanged MAE (14.242 versus 14.241 events/h). Among these three configurations, RF using segmented P achieved the lowest MAE in the 109-participant cohort, while SGD using segmented CF+P achieved the lowest MAE in the 105-participant cohort. In the reduced cohort, the median-AHI baseline MAE was 14.186 events/h for the unsegmented representation and 14.206 events/h for the segmented representation. SVR using unsegmented CF and SGD using segmented CF+P retained MAE point estimates below their respective baselines, whereas RF using segmented P had a slightly higher MAE than the segmented baseline.

## 4. Discussion

The present study evaluated the usefulness of craniofacial measurements and speech-derived acoustic features for noninvasive AHI estimation and binary screening using both unsegmented and temporally segmented speech representations. Overall, craniofacial information provided the most consistent predictive contribution across the evaluated tasks, whereas acoustic features alone showed limited discriminative performance for binary classification. The competitive performance achieved using craniofacial measurements is consistent with previous studies reporting the relevance of facial morphology for OSA assessment [8,11,13].

For AHI estimation, the best unsegmented result was obtained using craniofacial features alone with SVR (MAE = 14.537 events/h), whereas the lowest segmented MAE was achieved using continuous-speech features with RF (MAE = 14.242 events/h). These findings indicate that the relative contribution of craniofacial and acoustic information depended on the representation and regression model. Importantly, the segmented representation did not produce a uniform improvement across feature configurations. Although the best segmented MAE was numerically lower than the best unsegmented MAE, several segmented configurations remained close to or above their corresponding cohort-specific baseline values. Therefore, the present results do not support a general conclusion that temporal segmentation improves AHI estimation.

For binary classification, the unsegmented CF configuration achieved the highest AUC-ROC (0.766), whereas in the segmented dataset the highest AUC-ROC was obtained with CF+P (0.756). However, the segmented CF-only configuration reached a nearly identical AUC-ROC of 0.753, indicating that the addition of continuous-speech features did not provide a clear discriminative advantage. In contrast, feature sets based exclusively on acoustic information yielded AUC-ROC values close to chance level in both representations. Craniofacial measurements may capture morphological characteristics associated with OSA [8,11,13], whereas speech-derived acoustic features provide a functional representation of voice and speech characteristics that have also been associated with OSA [10,14,15]. Therefore, the present results support a more cautious interpretation of the contribution of speech-derived features: although they may provide complementary information in specific model and feature combinations, they did not consistently improve classification beyond craniofacial information alone.

A notable pattern across the classification results was the imbalance between sensitivity and specificity. Several of the selected models achieved high sensitivity, particularly those incorporating craniofacial information, but this was accompanied by comparatively low specificity. For example, in the unsegmented dataset, CF+P reached a sensitivity of 0.920 but a specificity of 0.376, while CF+V reached a sensitivity of 0.911 with a specificity of 0.388. A similar pattern was observed in the segmented dataset, where CF+P achieved a sensitivity of 0.931 but a specificity of 0.438.

This behavior indicates that the models tended to favor identification of participants in the positive screening class at the cost of a higher false-positive rate. Such a trade-off may be acceptable in a preliminary screening context, where minimizing missed cases can be clinically desirable; however, the low specificity limits the suitability of these models as stand-alone diagnostic tools. Accordingly, the present findings should be interpreted in the context of screening support rather than definitive OSA diagnosis.

Some model–feature combinations yielded AUC-ROC values close to or below 0.5, particularly when using exclusively acoustic features. An AUC-ROC near 0.5 indicates little ability to discriminate between the positive and negative screening classes, whereas values slightly below 0.5 should not necessarily be interpreted as evidence of a systematically inverse association. In the present study, these values occurred only for specific combinations of algorithms and feature sets and are more plausibly indicative of limited discriminatory information and variability associated with the relatively small sample. These findings further support the conclusion that the evaluated acoustic features alone did not provide robust information for binary screening.

Because multiple algorithms and feature configurations were compared in a relatively small cohort, the performance of the selected best-performing models may still be subject to model-selection optimism. The nested cross-validation framework used in this study reduces bias associated with hyperparameter tuning by separating model selection from external performance evaluation [18]. However, it does not completely remove the uncertainty introduced by comparing many candidate models and feature representations within a limited sample. Therefore, the reported performance estimates, particularly those corresponding to the highest-performing configurations, should be interpreted cautiously and validated in larger independent cohorts.

The sensitivity analysis suggested that the broad classification pattern observed in the primary analysis was not driven primarily by the four participants with the highest AHI values. Across the selected configurations, those incorporating craniofacial information retained AUC-ROC estimates close to 0.75, whereas the exclusively acoustic configuration remained close to 0.58. These findings support the consistency of the classification performance observed for configurations incorporating craniofacial features within this cohort. However, they do not establish an independent benefit of multimodal integration, because the selected configurations also differed in algorithm and representation.

Regression findings were more dependent on cohort composition. Excluding the four participants with the highest AHI values reduced MAE for SVR with unsegmented CF and SGD with segmented CF+P, but this reduction should be interpreted in the context of the altered outcome distribution. Lower error in the reduced cohort does not, by itself, demonstrate improved predictive capability. Performance relative to the corresponding baseline was particularly informative: RF with segmented P retained an almost unchanged MAE but no longer achieved a lower MAE than the median-AHI baseline. Conversely, SVR with unsegmented CF and SGD with segmented CF+P retained MAE point estimates below their respective baselines. The change in the configuration achieving the lowest MAE further indicates that the identity of the best-performing regressor was sensitive to cohort composition.

Overall, these findings qualify the evidence for acoustic and multimodal prediction rather than establishing a uniformly superior modality. The sensitivity analysis was restricted to six previously selected configurations and used a subset of the primary cohort. It therefore assesses the dependence of selected findings on the inclusion of the four highest AHI values rather than providing independent validation or identifying the best configuration across the full experimental design. The comparisons were descriptive and do not establish statistical equivalence between cohorts or significant superiority between models.

BMI represents a potential source of confounding when interpreting the contribution of craniofacial and acoustic features. Increased body mass is a well-established risk factor for OSA and may influence upper-airway structure and collapsibility [3,19]. Consequently, BMI-related anatomical variation may partly overlap with information captured by some of the craniofacial measurements. In the present study, BMI and the other clinical variables were included for cohort characterization but were not incorporated as predictors in the machine-learning models. Consequently, the current analysis cannot determine to what extent the predictive information attributed to craniofacial morphology is independent of BMI. Future studies should evaluate the incremental value of craniofacial and acoustic features after adjustment for BMI and other established clinical risk factors.

The craniofacial measurements derived from facial photographs should also be interpreted in light of methodological limitations inherent to image-based anthropometry. Measurements obtained from two-dimensional photographs may be affected by camera perspective, lens characteristics, head positioning, landmark localization, and image calibration [20]. Consequently, these variables should be regarded as photographic craniofacial descriptors rather than direct measurements of underlying skeletal or internal anatomical structures.

In particular, measurements derived from surface landmarks cannot be interpreted as true volumetric measurements of internal cranial compartments. Accordingly, the photographic n–a–t area × frontal-landmark width proxy should be interpreted as a geometric descriptor derived from facial landmarks rather than as direct cranial-fossa volumetry. Although physical scaling was performed using QR-based calibration, this descriptor remains a two-dimensional photographic proxy rather than a direct anatomical volume. Furthermore, formal intra- and inter-observer reliability of manual landmark refinement was not assessed, and reviewer blinding to the sleep-study results was not formally documented. These factors should be considered when interpreting the craniofacial descriptors and should be addressed in future validation studies.

Among the studies reviewed in the Introduction, the work of Espinoza-Cuadros et al. [11] provides one of the closest methodological comparisons with the present study because it also investigated facial and speech-derived information for OSA assessment. However, several differences limit direct numerical comparison. Espinoza-Cuadros et al. evaluated a male Caucasian cohort and incorporated clinical variables together with acoustic or craniofacial information, whereas the present study included both women and men and evaluated craniofacial and acoustic descriptors without incorporating clinical variables as model predictors. In addition, the two studies used different facial representations and machine-learning evaluation procedures. Espinoza-Cuadros et al. reported a minimum MAE of 11.97 events/h for AHI estimation, whereas the lowest MAE obtained in the primary cohort of the present study was 14.242 events/h. Given the differences in population, predictor sets, modeling strategy, and validation procedures, these values should not be interpreted as a direct comparison of model performance.

Other studies have also demonstrated the potential of facial information for OSA assessment, although using substantially different representations. Balaei et al. [13] investigated OSA detection from facial images, while He et al. [8] and Islam et al. [21] applied deep-learning approaches to craniofacial images or facial depth information. Unlike these image-based approaches, the present study used a limited set of explicitly defined photographic craniofacial descriptors derived from facial reference points. The present findings therefore provide complementary evidence that relatively compact craniofacial representations can contain information relevant to OSA screening, while also highlighting the methodological limitations associated with measurements derived from two-dimensional photographs.

Speech-based OSA assessment has likewise been investigated using a variety of acoustic representations. Goldshtein et al. [22] analyzed speech signals for automatic OSA detection, while Perero-Codosero et al. [14] used deep neural-network embeddings and domain-adversarial training. Ding et al. [10] evaluated speech features for OSA severity assessment, and Pang et al. [15] focused on high-frequency speech components. These approaches differ substantially from the conventional acoustic descriptors evaluated here, which were primarily based on fundamental frequency, harmonicity, jitter, and shimmer. The relatively weak classification performance obtained from the acoustic-only configurations in the present study should therefore not be interpreted as evidence that speech is generally uninformative for OSA screening. Rather, it suggests that the particular acoustic representation evaluated here did not provide robust discrimination in this cohort.

Overall, the present study supports the potential of noninvasive craniofacial and speech-derived information for machine-learning-based OSA screening, while also highlighting important methodological limitations. Craniofacial descriptors provided the most consistent predictive contribution across tasks, whereas the value of acoustic features depended on the representation, model, and prediction setting. The study was conducted in a relatively limited single-center cohort, and several aspects require further validation, including external evaluation in independent populations, assessment of photographic landmark reproducibility, control of image-acquisition geometry, and evaluation of clinical confounding factors such as BMI. In addition, the sensitivity–specificity trade-off observed in classification indicates that the proposed models should be regarded as preliminary screening tools rather than replacements for standard diagnostic procedures. Future studies should therefore focus on larger and more diverse cohorts, improved acoustic representations, robust multimodal integration strategies, and clinically relevant operating thresholds.

## 5. Conclusions

This study evaluated noninvasive machine-learning approaches for AHI estimation and binary OSA screening using photographic craniofacial descriptors and speech-derived acoustic features in both unsegmented and temporally segmented representations. Across the evaluated tasks, craniofacial information provided the most consistent predictive contribution, whereas acoustic-only configurations showed limited discriminative performance for binary classification.

For AHI estimation, the lowest MAE in the primary cohort was obtained using segmented continuous-speech features with RF (14.242 events/h), although performance varied across feature configurations and segmentation did not provide a uniform advantage. For classification, the highest AUC-ROC values were 0.766 for unsegmented CF and 0.756 for segmented CF+P, with the segmented CF-only configuration achieving a similar AUC-ROC of 0.753. These findings indicate that adding acoustic information did not consistently improve discrimination beyond craniofacial information alone.

Several selected classifiers showed high sensitivity but comparatively low specificity, which limits their use as stand-alone diagnostic models and supports interpretation of the proposed approach as preliminary screening support. The sensitivity analysis further indicated that the main classification pattern was not primarily driven by the four participants with the highest AHI values, although regression performance was more sensitive to cohort composition.

Overall, the results support further investigation of photographic craniofacial descriptors as noninvasive predictors for OSA screening, while the contribution of the acoustic features evaluated here appears to depend on the representation, model, and prediction task. External validation in larger and more diverse cohorts, assessment of photographic measurement reproducibility, evaluation of clinical confounding factors, and investigation of alternative acoustic and multimodal representations are needed before clinical application.

## Figures and Tables

**Figure 1:**
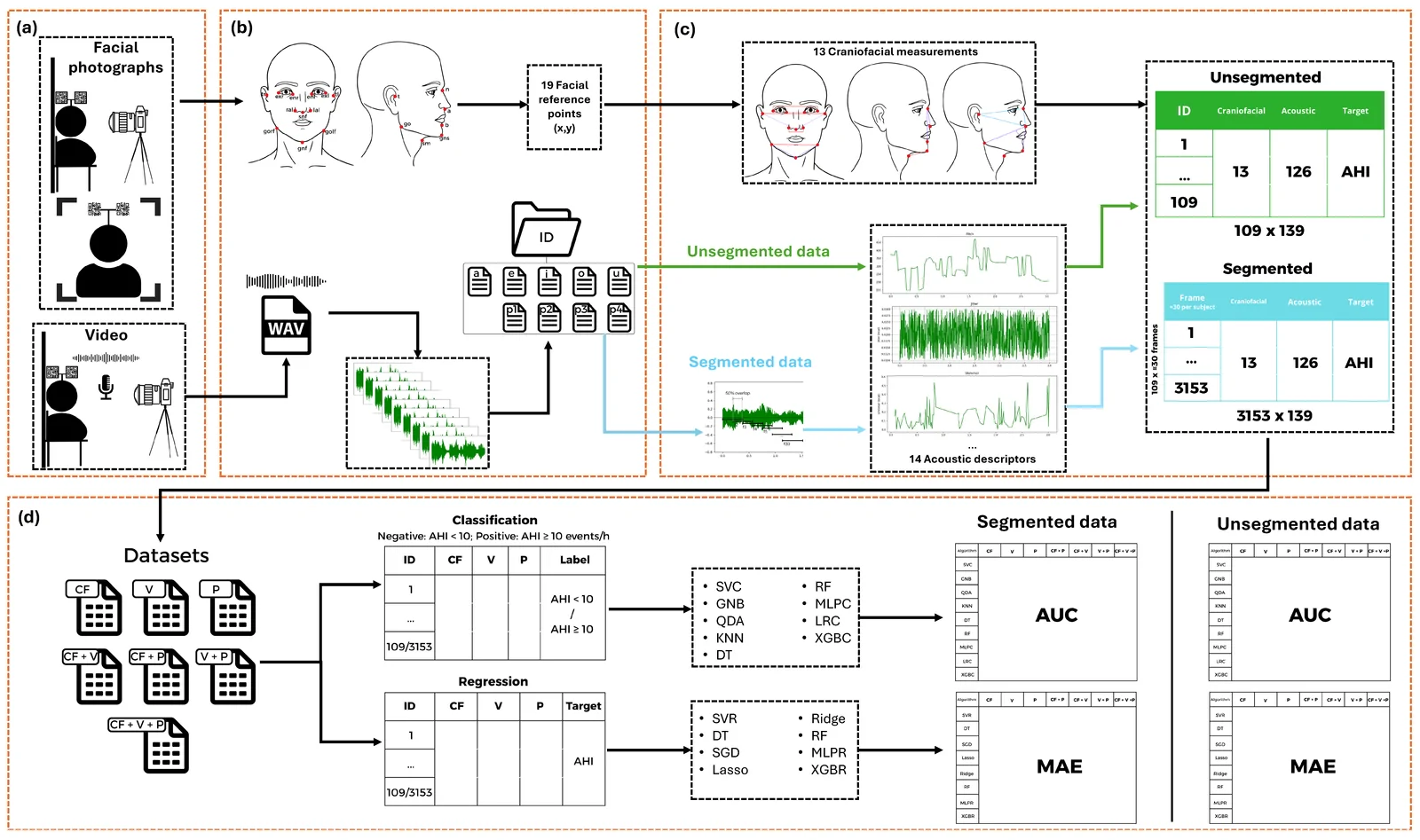
Overview of the proposed methodology. (**a**) Data acquisition, including facial photographs and speech recordings. (**b**) Automatic facial landmark detection followed by specialist validation. (**c**) Feature extraction from craniofacial measurements and speech signals using both unsegmented and segmented speech representations. (**d**) Construction of the experimental feature configurations and development of the machine learning models for AHI regression and OSA classification.

**Figure 2:**
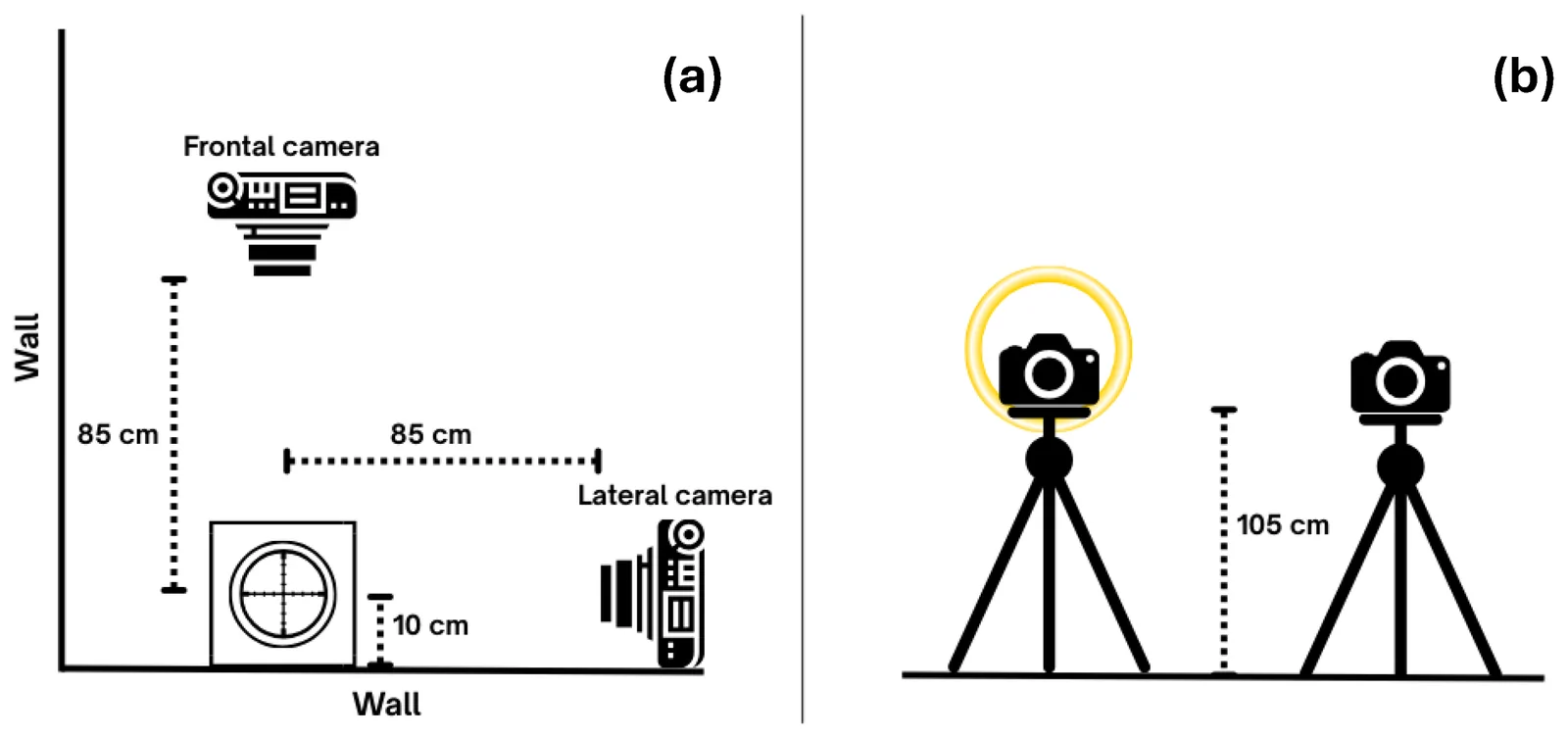
Acquisition setup. (**a**) shows the top view of the installed acquisition equipment. (**b**) shows the height of the lateral and frontal cameras.

**Figure 3:**
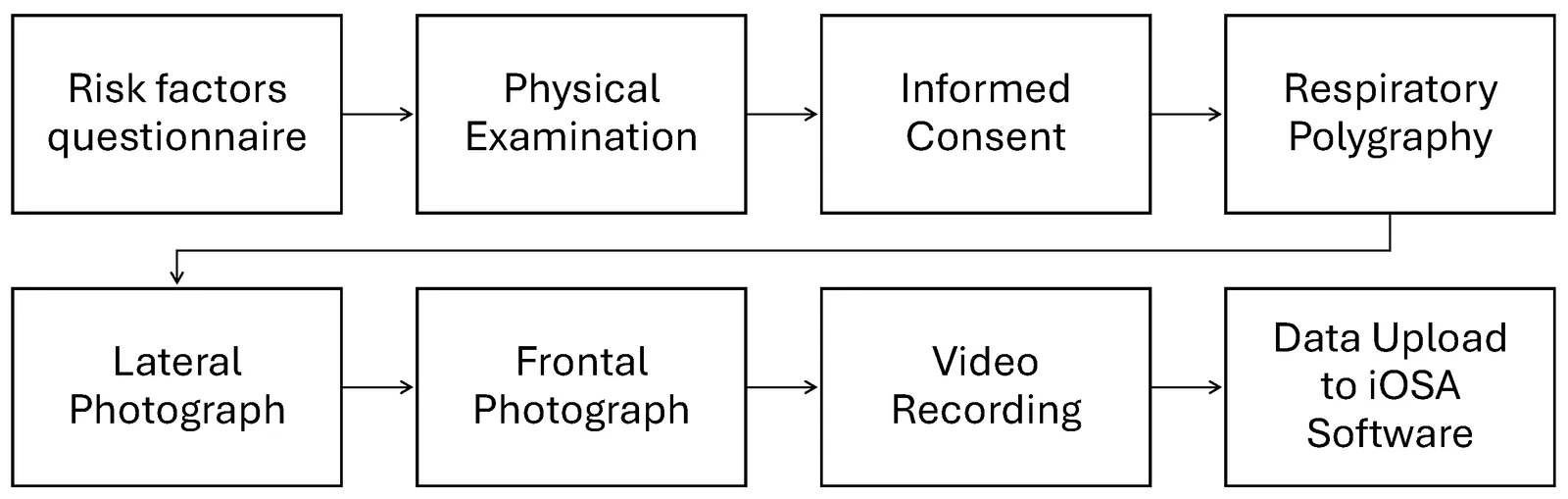
Data acquisition process flow. The entire acquisition process was carried out by medical specialists at the CMNO.

**Figure 4:**
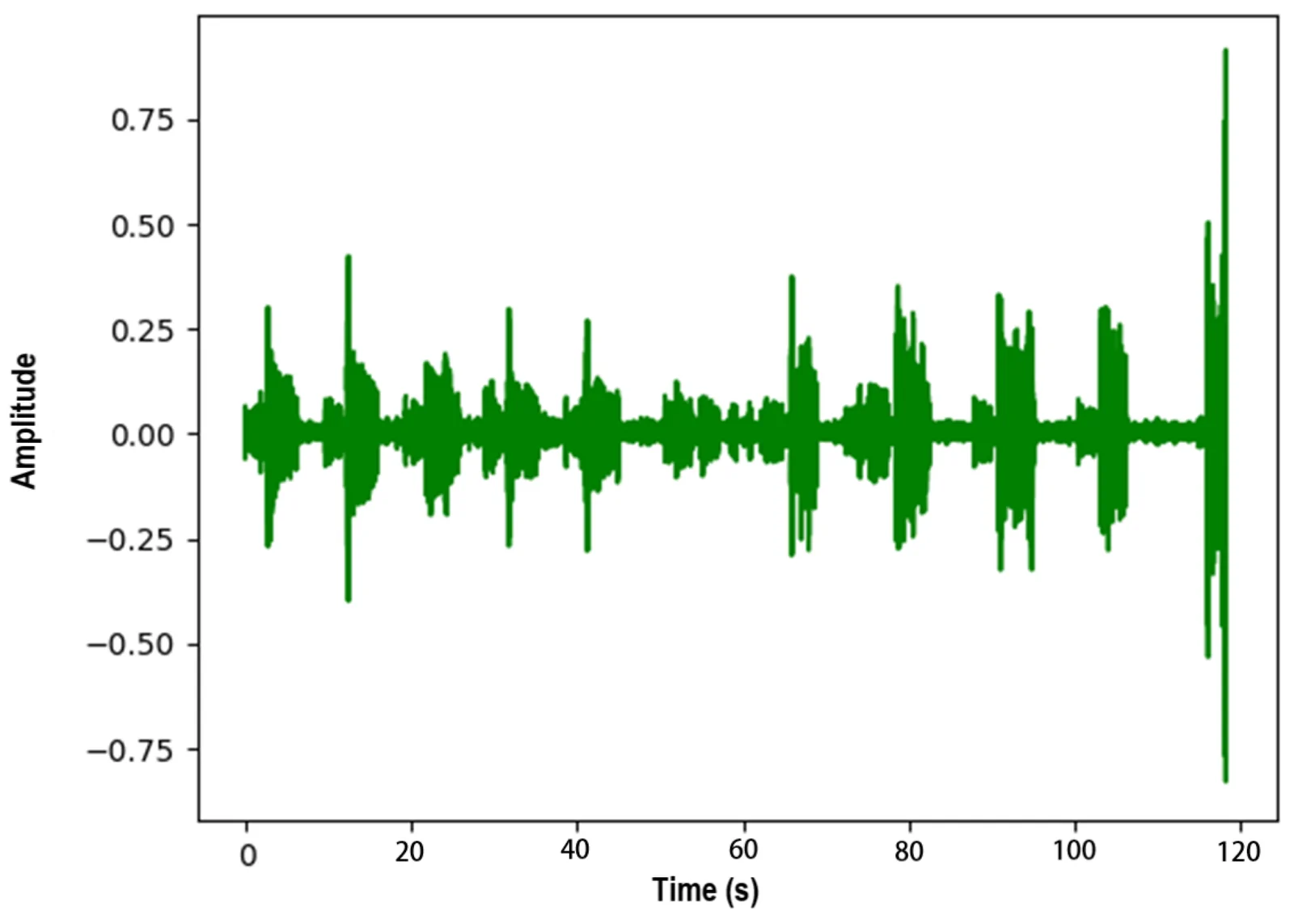
Raw speech signal. This visualization includes both vocalizations (vowels and phrases) and beeps, but they are not individually identified.

**Figure 5:**
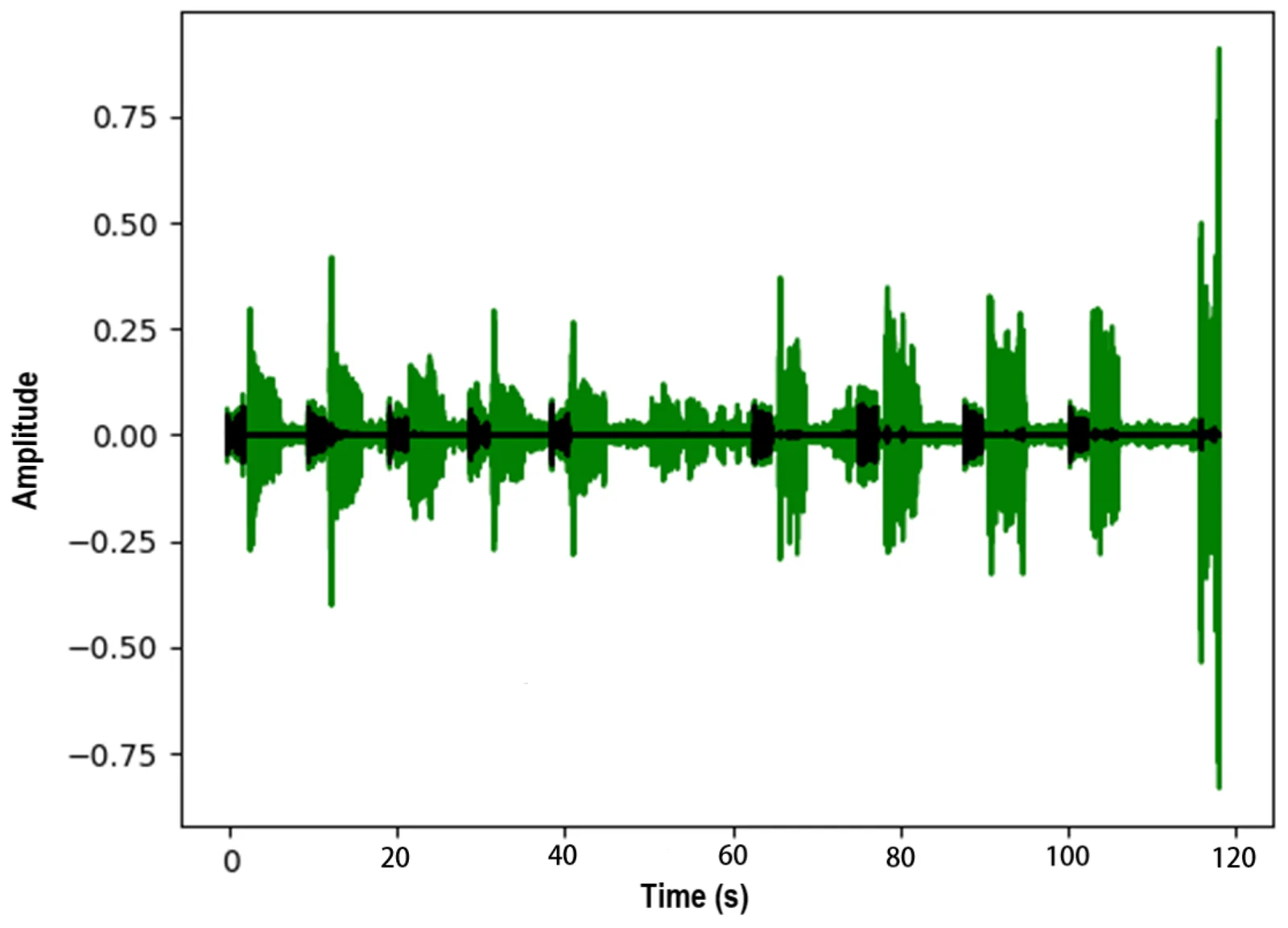
Speech signal and detected synchronization beeps. The raw signal is shown in light green, whereas the filtered signal highlighting the synchronization beeps is shown in dark green.

**Figure 6:**
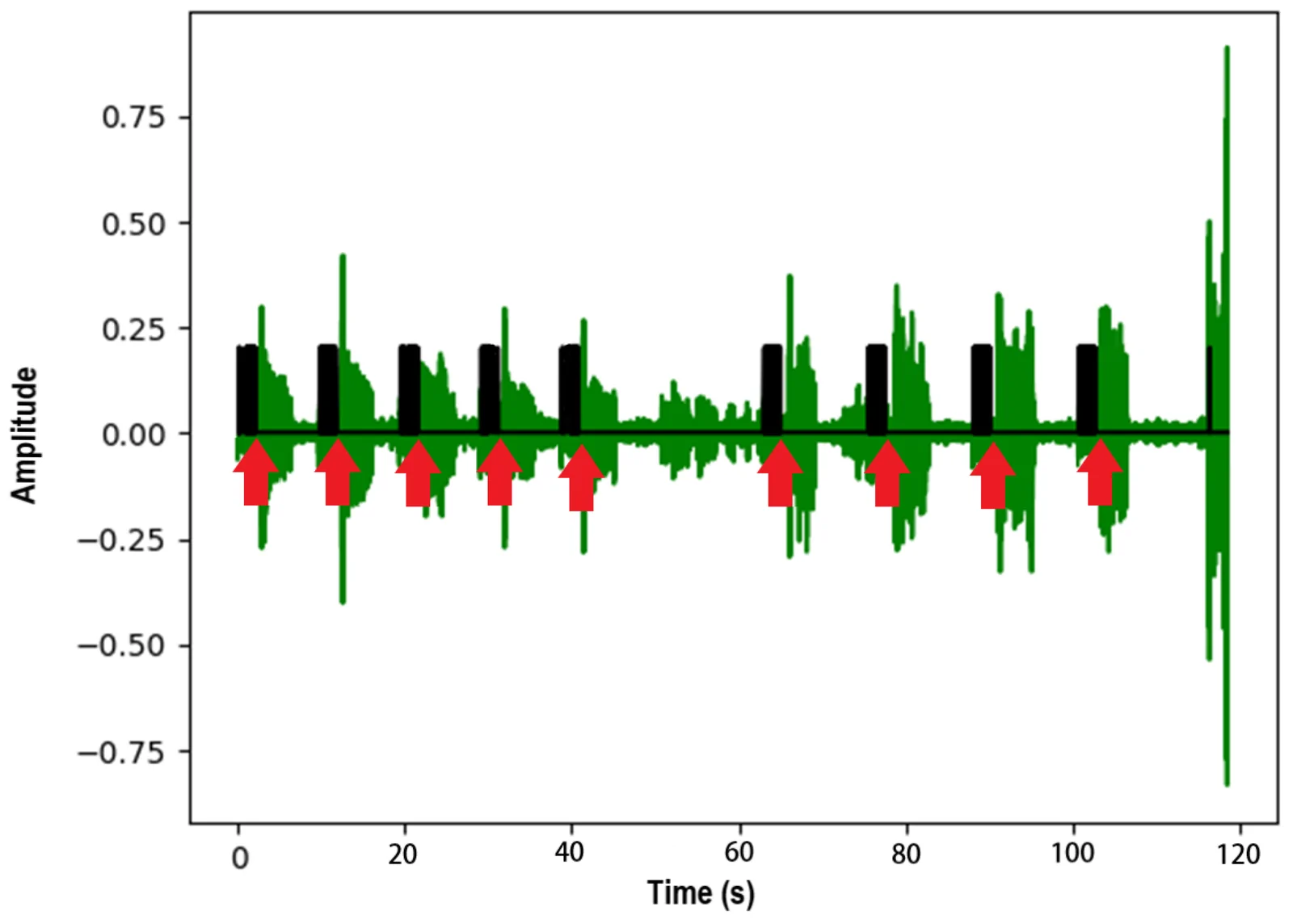
Speech signal showing the segmentation points used to isolate the nine speech segments. The intervals indicated by the red arrows are removed during preprocessing. The light green color indicates the subject’s vocalizations, while the dark green color indicates the instructions provided for the subject during the instructional video. These were the removed portions.

**Figure 7:**
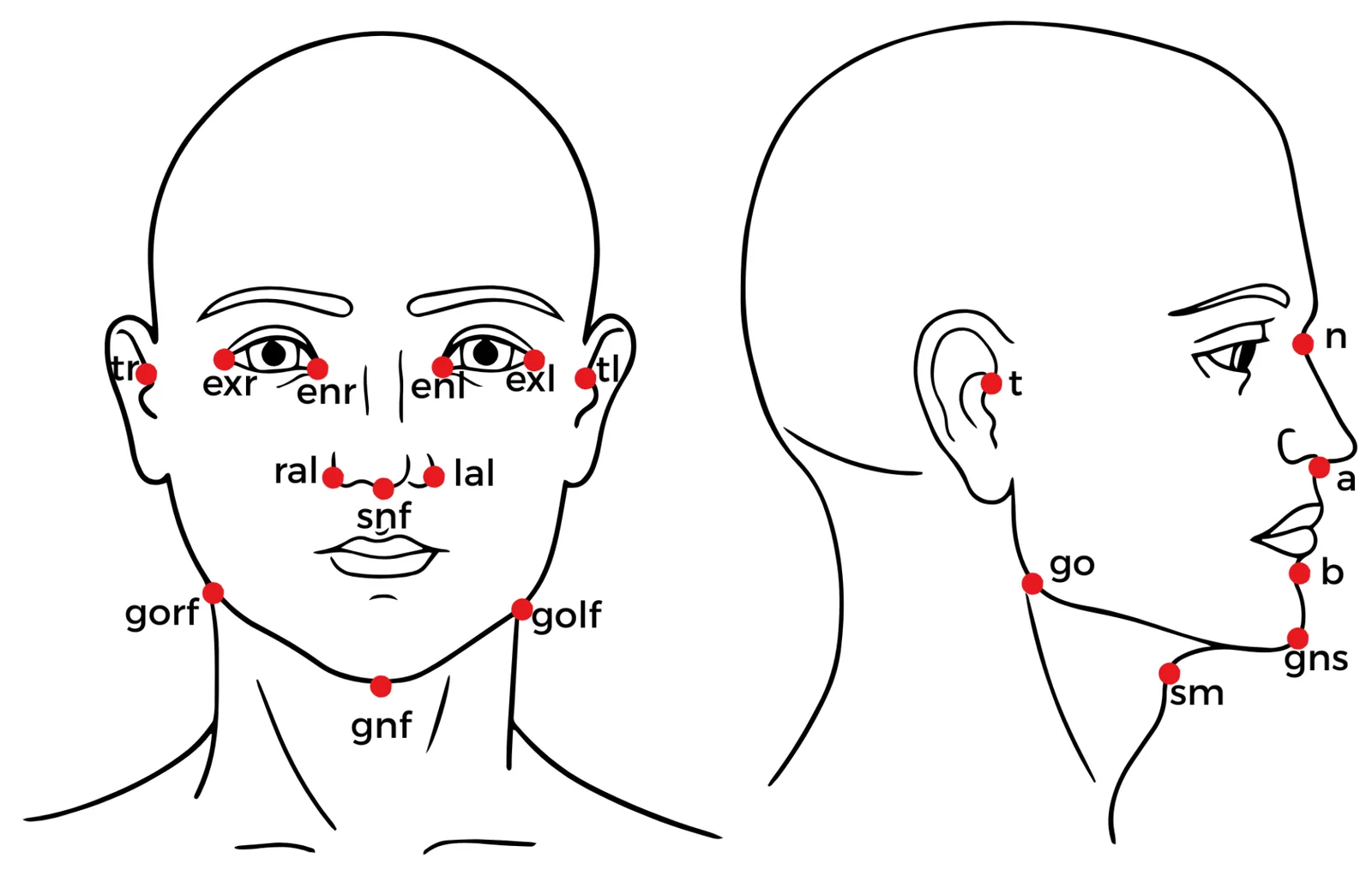
Schematic locations of the photographic facial reference points used in this study: frontal view (**left**) and lateral view (**right**). Landmark labels correspond to those defined in Table 1.

**Figure 8:**
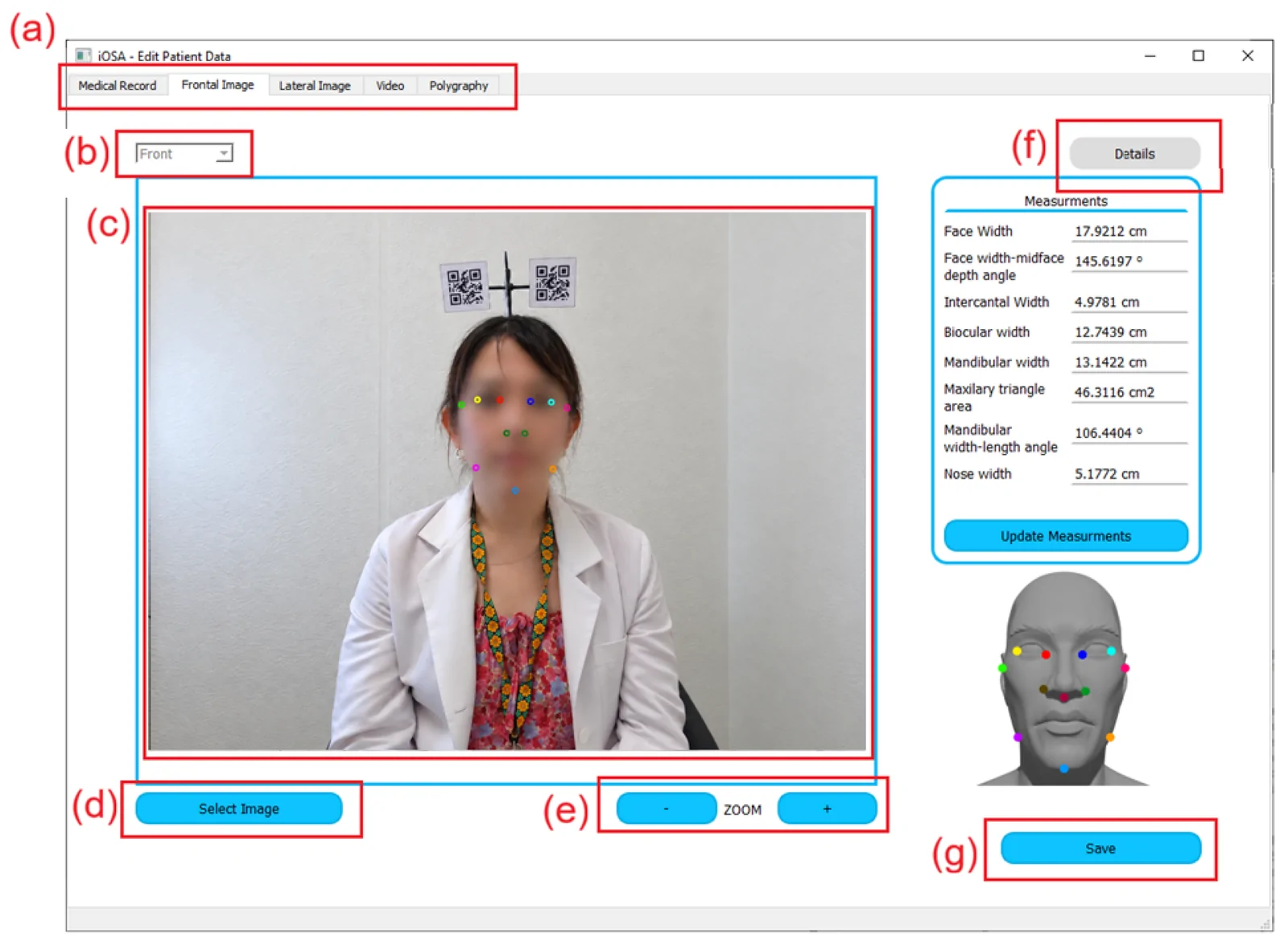
*iOSA* interface used for visualization and manual refinement of facial landmarks. The facial image was blurred for anonymization, while landmark positions were retained for illustrative purposes. (**a**) Menu; (**b**) frontal or lateral image selector; (**c**) image display with facial landmarks; (**d**) patient image selector; (**e**) zoom controls; (**f**) measurements calculated from facial reference points; and (**g**) save button.

**Table 1:** Description of the facial reference points.

#	Label	Description	Image
1	tr	Right tragion	Frontal
2	tl	Left tragion	Frontal
3	exr	Right exocanthion	Frontal
4	exl	Left exocanthion	Frontal
5	enr	Right endocanthion	Frontal
6	enl	Left endocanthion	Frontal
7	ral	Right alar	Frontal
8	lal	Left alar	Frontal
9	snf	Subnasale frontal	Frontal
10	gorf	Right gonion	Frontal
11	golf	Left gonion	Frontal
12	gnf	Frontal gnathion	Frontal
13	t	Lateral tragion	Lateral
14	n	Nasion	Lateral
15	a	Point A	Lateral
16	b	Point B	Lateral
17	go	Lateral gonion	Lateral
18	gns	Lateral gnathion	Lateral
19	sm	Submental point	Lateral

Note: The landmark labels used by *iOSA* denote photographic surface reference points and should not be interpreted as skeletal cephalometric landmarks with similar nomenclature.

**Table 2:** Photographic craniofacial descriptors calculated by the *iOSA* software.

#	Measurement	Reference Points	Unit
1	Frontal width angle	ral–snf–lal	degree
2	Photographic n–b–a angle	n–b–a	degree
3	Mandibular width angle	gorf–gnf–golf	degree
4	Lateral n–go–gns angle	n–go–gns	degree
5	Lateral a–go–gns angle	a–go–gns	degree
6	Horizontal submental distance	sm–gns	cm
7	Horizontal intercantal width	enr–enl	cm
8	Horizontal nasal width	ral–lal	cm
9	Horizontal biocular width	exr–exl	cm
10	Horizontal frontal width	tr–tl	cm
11	Horizontal mandibular width	gorf–golf	cm
12	Frontal triangular area	tr–snf–tl	cm^2^
13	Photographic n–a–t area × frontal-landmark width proxy	n–a–t; tr–tl	cm^3^

Reference-point labels are defined in Table 1. The cm^3^ unit of the final descriptor reflects the dimensional result of the software calculation (area × width) and should not be interpreted as a directly measured anatomical volume.

**Table 3:** Extracted acoustic features.

#	Feature	Variable
1	Fundamental Frequency Mean	meanF0
2	Fundamental Frequency Std. Deviation	stdevF0
3	Harmonic to Noise Ratio	HNR
4	Local Jitter	localJitter
5	Absolute Local Jitter	localAbsoluteJitter
6	Relative Average Perturbation Jitter	rapJitter
7	PPQ5 Jitter	ppq5Jitter
8	Peak to Peak deviation Jitter	ddpJitter
9	Local Shimmer	localShimmer
10	Local Shimmer in db	localdbShimmer
11	APQ3 Shimmer	apq3Shimmer
12	APQ5 Shimmer	apq5Shimmer
13	APQ11 Shimmer	apq11Shimmer
14	dda Shimmer	ddaShimmer

**Table 4:** Demographic and clinical characteristics of the study population.

Characteristic	Overall (n=109)	AHI <10 (n=34)	AHI ≥10 (n=75)
Age, years	53.9±14.0	46.6±15.5	57.2±12.1
BMI, kg/m^2^	36.27±8.52	34.67±8.88	36.99±8.31
Neck circumference, cm	42.36±5.39	39.41±3.38	43.69±5.62
Oxygen saturation, %	94.23±2.44	94.62±2.13	94.05±2.56
Hypertension, *n* (%)	77 (70.6)	18 (52.9)	59 (78.7)
Current smoking, *n* (%)	26 (23.9)	4 (11.8)	22 (29.3)
Former smoker, *n* (%)	24 (22.0)	2 (5.9)	22 (29.3)
Snoring, *n* (%)	100 (91.7)	28 (82.4)	72 (96.0)
Witnessed apneas, *n* (%)	93 (85.3)	23 (67.6)	70 (93.3)
Epworth Sleepiness Scale, points	10.32±5.08	7.88±5.38	11.43±4.56
Mallampati class I, *n* (%)	3/106 (2.8)	0/32 (0.0)	3/74 (4.1)
Mallampati class II, *n* (%)	22/106 (20.8)	12/32 (37.5)	10/74 (13.5)
Mallampati class III, *n* (%)	72/106 (67.9)	18/32 (56.3)	54/74 (73.0)
Mallampati class IV, *n* (%)	9/106 (8.5)	2/32 (6.3)	7/74 (9.5)

Continuous variables are presented as mean ± SD and categorical variables as *n* (%). Age was calculated at the date of the sleep study. BMI was calculated from measured weight and height. Mallampati percentages were calculated using available data only; Mallampati information was unavailable for three participants. AHI: Apnea–Hypopnea Index; BMI: body mass index; SD: standard deviation.

**Table 5:** Distribution of participants according to AHI range, binary screening class, and sex.

AHI Range/Screening Class	Women, *n*	Men, *n*	Total, *n* (%)
AHI <5 events/h	14	8	22 (20.2)
5≤ AHI <10 events/h	8	4	12 (11.0)
Negative screening class (AHI <10)	22	12	34 (31.2)
10≤ AHI <15 events/h	1	7	8 (7.3)
15≤ AHI <30 events/h	20	13	33 (30.3)
AHI ≥30 events/h	11	23	34 (31.2)
Positive screening class (AHI ≥10)	32	43	75 (68.8)
Total	54	55	109 (100)

AHI ranges are shown for descriptive purposes and should be distinguished from the binary classes used for machine learning. For the classification experiments, AHI <10 events/h was defined as the negative screening class and AHI ≥10 events/h as the positive screening class. AHI: Apnea–Hypopnea Index.

**Table 6:** Regression performance for the unsegmented dataset by feature set.

Feature Set	Best Model	MAE (95% CI), Events/h
**CF**	**SVR**	**14.537 (12.239–17.059)**
V	SVR	16.328 (13.977–19.000)
P	SVR	16.085 (13.696–18.691)
V+P	SVR	16.040 (13.626–18.733)
CF+V	RF	15.667 (13.592–17.686)
CF+P	SGD	15.327 (12.956–17.848)
CF+V+P	RF	15.700 (13.625–17.828)
Median-AHI baseline	—	15.915 (13.336–18.643)

All performance measures were computed at the participant level. The median-AHI baseline corresponds to predicting the median AHI of the corresponding outer-training participants.

**Table 7:** Regression performance for the segmented dataset by feature set.

Feature Set	Best Model	MAE (95% CI), Events/h
CF	SVR	14.713 (12.349–17.072)
V	Lasso	15.934 (13.730–18.417)
**P**	**RF**	**14.242 (12.296–16.290)**
V+P	XGBR	15.787 (13.795–18.208)
CF+V	XGBR	15.150 (12.953–17.477)
CF+P	SGD	14.761 (12.336–17.615)
CF+V+P	XGBR	15.116 (13.000–17.427)
Median-AHI baseline	—	15.871 (13.257–18.673)

All performance measures were computed at the participant level. The median-AHI baseline corresponds to predicting the median AHI of the corresponding outer-training participants.

**Table 8:** Classification performance for the unsegmented dataset by feature set.

Feature Set	Best Model	AUC-ROC (95% CI)	Balanced Accuracy	Sensitivity	Specificity	Accuracy
**CF**	**MLPC**	**0.766 (0.671–0.846)**	**0.673**	**0.829**	**0.518**	**0.732**
V	MLPC	0.571 (0.476–0.663)	0.551	0.749	0.353	0.626
P	RF	0.550 (0.450–0.647)	0.515	0.772	0.259	0.612
V+P	MLPC	0.578 (0.483–0.668)	0.532	0.767	0.297	0.620
CF+V	RF	0.704 (0.599–0.802)	0.649	0.911	0.388	0.748
CF+P	RF	0.685 (0.567–0.793)	0.648	0.920	0.376	0.750
CF+V+P	RF	0.678 (0.569–0.784)	0.638	0.911	0.365	0.740
Majority-class baseline	—	0.500 (0.500–0.500)	0.500	1.000	0.000	0.688

All performance measures were computed at the participant level. The majority-class baseline corresponds to predicting the positive screening class for all outer-test participants.

**Table 9:** Classification performance for the segmented dataset by feature set.

Feature Set	Best Model	AUC-ROC (95% CI)	Balanced Accuracy	Sensitivity	Specificity	Accuracy
CF	RF	0.753 (0.655–0.841)	0.684	0.916	0.453	0.772
V	DT	0.521 (0.450–0.589)	0.488	0.724	0.253	0.577
P	XGBC	0.568 (0.468–0.662)	0.547	0.788	0.306	0.638
V+P	LRC	0.510 (0.416–0.599)	0.485	0.656	0.315	0.550
CF+V	MLPC	0.751 (0.651–0.838)	0.679	0.847	0.512	0.742
**CF+P**	**RF**	**0.756 (0.651–0.849)**	**0.684**	**0.931**	**0.438**	**0.777**
CF+V+P	LRC	0.749 (0.652–0.836)	0.681	0.827	0.535	0.736
Majority-class baseline	—	0.500 (0.500–0.500)	0.500	1.000	0.000	0.688

All performance measures were computed at the participant level. The majority-class baseline corresponds to predicting the positive screening class for all outer-test participants.

**Table 10:** Sensitivity analysis comparing the six selected configurations between the primary cohort of 109 participants and the reduced cohort of 105 participants. Values are point estimates with 95% confidence intervals.

Model	Feature Set	Representation	109 Participants	105 Participants
Classification: AUC-ROC				
MLPC	CF	Unsegmented	0.766 (0.671–0.846)	0.750 (0.655–0.829)
MLPC	V+P	Unsegmented	0.578 (0.483–0.668)	0.577 (0.491–0.671)
RF	CF+P	Segmented	0.756 (0.651–0.849)	0.755 (0.652–0.849)
Regression: MAE, events/h				
SVR	CF	Unsegmented	14.537 (12.239–17.059)	13.417 (11.522–15.415)
RF	P	Segmented	14.242 (12.296–16.290)	14.241 (12.139–16.267)
SGD	CF+P	Segmented	14.761 (12.336–17.615)	13.120 (11.027–15.316)

CF: craniofacial features; V: sustained-vowel features; P: continuous-speech features. The majority-class baseline had an AUC-ROC of 0.500 in both cohorts. Median-AHI baseline MAE values for the unsegmented and segmented representations were, respectively, 15.915 and 15.871 events/h with 109 participants, and 14.186 and 14.206 events/h with 105 participants.

## Data Availability

The datasets generated and analyzed during the current study are not publicly available at the time of publication due to ethical restrictions and the provisions of the Mexican General Law on the Protection of Personal Data. Data availability will be evaluated after appropriate anonymization and approval by the corresponding ethics committee.

## Supplementary Materials

The following supporting information can be downloaded at https://www.mdpi.com/article/10.3390/life16101748/s1, Table S1: Demographic and clinical characteristics; Table S2: Missing data after verification; Table S3: Availability of segmented instances; Table S4: Hyperparameter search settings; Table S5: Regression MAE—Unsegmented features; Table S6: Regression RMSE—Unsegmented features; Table S7: Regression MAE—Segmented features; Table S8: Regression RMSE—Segmented features; Table S9: Classification AUC-ROC—Unsegmented features; Table S10: Classification AUC-ROC—Segmented features.

## Abbreviations

The following abbreviations are used in this manuscript:

AHI	Apnea–Hypopnea Index
CF	Craniofacial Measurements
CMNO	Western National Medical Center
CNN	Convolutional Neural Network
DT	Decision Trees
ECG	Electrocardiography
EEG	Electroencephalography
EMG	Electromyography
EOG	Electrooculography
GNB	Gaussian Naive Bayes
IMSS	Mexican Social Security Institute
KNN	K-Nearest Neighbors
LRC	Logistic Regression Classifier
MAE	Mean Absolute Error
ML	Machine Learning
MLPC	Multilayer Perceptron Classifier
MLPR	Multilayer Perceptron Regressor
OSA	Obstructive Sleep Apnea
PSG	Polysomnography
QDA	Quadratic Discriminant Analysis
RF	Random Forests
RP	Respiratory Polygraphy
RSCV	Randomized Search Cross-Validation
SECIHTI	Secretariat of Science, Humanities, Technology and Innovation
SGD	Stochastic Gradient Descent
SpO_2_	Blood Oxygen Saturation
SVC	Support Vector Classifier
SVR	Support Vector Regressor
