# Supplementary material for: Machine Learning-Based Screening for Obstructive Sleep Apnea Using Photographic Craniofacial and Speech-Derived Acoustic Features
Source: Life (Basel). 2026 Oct 18;16(10):1748. doi: 10.3390/life16101748 (PMC13705782; doi:10.3390/life16101748)
Supplement: Supplementary file 1 [file life-16-01748-s001.zip › life-4460177-supplementary.pdf]

Supplementary Materials

Machine Learning-Based Screening for Obstructive Sleep Apnea Using Photographic Craniofacial and Speech-Derived Acoustic Features

Marissa López-Zaldivar, Sulema Torres-Ramos, Israel Román-Godínez,
Daniel Hernández-Gordillo, Aurora S. Vázquez-Jiménez and Ricardo A. Salido-Ruiz

Contents

Table S1. Demographic and clinical characteristics 2
Table S2. Missing data after verification 4
Table S3. Availability of segmented instances 5
Table S4. Hyperparameter search settings 6
Table S5. Regression MAE – Unsegmented features 8
Table S6. Regression RMSE – Unsegmented features 9
Table S7. Regression MAE – Segmented features 10
Table S8. Regression RMSE – Segmented features 11
Table S9. Classification AUC-ROC – Unsegmented features 12
Table S10. Classification AUC-ROC – Segmented features 13

Demographic and clinical characteristics

Supplementary Table S1 presents the complete demographic and clinical characteristics of the 109 participants included in the primary analysis, both overall and stratified by the binary screening threshold: AHI < 10 events/h (n = 34) and AHI ≥ 10 events/h (n = 75). The table includes anthropometric measurements, vital signs, sleep-related symptoms, and relevant clinical characteristics. Continuous variables are summarized using both mean ± standard deviation and median [first–third quartiles], with the number of valid observations indicated for each variable and group. Categorical variables are presented as counts and percentages, calculated using the available observations within each column as the denominator.

Table S1: Complete demographic and clinical characteristics.

Characteristic	Overall (n = 109)	AHI < 10 (n = 34)	AHI ≥ 10 (n = 75)
AHI, events/h	24.55 ± 20.90; 21.50 [8.40–32.40] (n = 109)	4.48 ± 3.02; 4.65 [1.40–6.45] (n = 34)	33.65±19.09; 27.90 [20.95–41.15] (n = 75)
Age, years	53.90±14.04; 56.00 [43.00–64.00] (n = 109)	46.62±15.48; 46.00 [32.50–59.00] (n = 34)	57.20±12.06; 61.00 [48.50–66.00] (n = 75)
Height, m	1.64 ± 0.09; 1.63 [1.58–1.70] (n = 109)	1.62 ± 0.08; 1.61 [1.58–1.69] (n = 34)	1.65 ± 0.09; 1.63 [1.58–1.71] (n = 75)
Weight, kg	97.17±23.16; 94.00 [82.00–109.00] (n = 109)	91.29±23.88; 88.50 [75.25–104.75] (n = 34)	99.84±22.48; 97.00 [83.00–111.50] (n = 75)
BMI, kg/m^2	36.27 ± 8.52; 35.16 [29.35–41.80] (n = 109)	34.67 ± 8.88; 34.21 [26.76–40.58] (n = 34)	36.99 ± 8.31; 35.38 [31.05–42.73] (n = 75)
Neck circumference, cm	42.36 ± 5.39; 42.00 [39.00–44.00] (n = 109)	39.41 ± 3.38; 39.50 [37.00–42.00] (n = 34)	43.69 ± 5.62; 43.00 [40.00–45.00] (n = 75)
Heart rate, bpm	77.90±11.77; 78.00 [70.00–84.00] (n = 109)	78.53±11.96; 78.00 [70.00–84.75] (n = 34)	77.61±11.76; 78.00 [70.00–84.00] (n = 75)
Systolic blood pressure, mmHg	121.72 ± 11.65; 120.00 [115.00–129.00] (n = 108)	121.03 ± 11.36; 120.00 [116.00–128.00] (n = 33)	122.03 ± 11.84; 120.00 [115.00–129.50] (n = 75)
Diastolic blood pressure, mmHg	77.44 ± 6.73; 79.00 [72.00–81.00] (n = 108)	76.82 ± 7.32; 78.50 [70.50–80.00] (n = 34)	77.72 ± 6.48; 79.00 [73.25–81.00] (n = 74)
Respiratory rate, breaths/min	17.92 ± 1.24; 18.00 [18.00–18.00] (n = 108)	18.03 ± 1.42; 18.00 [18.00–18.75] (n = 34)	17.86 ± 1.15; 18.00 [18.00–18.00] (n = 74)
Oxygen saturation, %	94.23 ± 2.44; 95.00 [94.00–95.00] (n = 109)	94.62 ± 2.13; 95.00 [95.00–95.75] (n = 34)	94.05 ± 2.56; 95.00 [94.00–95.00] (n = 75)
Epworth Sleepiness Scale, points	10.32 ± 5.08; 11.00 [7.00–14.00] (n = 109)	7.88 ± 5.38; 7.00 [3.25–11.50] (n = 34)	11.43 ± 4.56; 12.00 [8.00–14.50] (n = 75)
Sex: Male	55 (50.5%)	12 (35.3%)	43 (57.3%)
Female	54 (49.5%)	22 (64.7%)	32 (42.7%)
Oxygen use: No	101 (92.7%)	32 (94.1%)	69 (92.0%)
Yes	8 (7.3%)	2 (5.9%)	6 (8.0%)
Current smoking: No	83 (76.1%)	30 (88.2%)	53 (70.7%)
Yes	26 (23.9%)	4 (11.8%)	22 (29.3%)
Former smoker: No	85 (78.0%)	32 (94.1%)	53 (70.7%)
Yes	24 (22.0%)	2 (5.9%)	22 (29.3%)
Snoring: No	9 (8.3%)	6 (17.6%)	3 (4.0%)
Yes	100 (91.7%)	28 (82.4%)	72 (96.0%)
Witnessed apneas: No	16 (14.7%)	11 (32.4%)	5 (6.7%)

Continued on next page

Supplementary Table S1 continued

**Characteristic**	**Overall (*n* = 109)**	**AHI < 10 (*n* = 34)**	**AHI ≥ 10 (*n* = 75)**
Yes	93 (85.3%)	23 (67.6%)	70 (93.3%)
Chronic fatigue: No	14 (12.8%)	9 (26.5%)	5 (6.7%)
Yes	95 (87.2%)	25 (73.5%)	70 (93.3%)
Hypertension: No	32 (29.4%)	16 (47.1%)	16 (21.3%)
Yes	77 (70.6%)	18 (52.9%)	59 (78.7%)
Mallampati class: I	3 (2.8%)	0 (0.0%)	3 (4.1%)
II	22 (20.8%)	12 (37.5%)	10 (13.5%)
III	72 (67.9%)	18 (56.2%)	54 (73.0%)
IV	9 (8.5%)	2 (6.2%)	7 (9.5%)

Notes. Age was calculated at the date of the sleep study. BMI was calculated from measured weight and height. Mallampati percentages exclude the three missing records. AHI = Apnea–Hypopnea Index; BMI = body mass index; SD = standard deviation; Q1 = first quartile; Q3 = third quartile.

Missing data after database verification

Supplementary Table S2 summarizes the availability of the clinical variables affected by missing or unrecoverable entries after database verification. For each variable, the table reports the numbers of available and missing observations and the reason for their treatment as missing. Systolic blood pressure, diastolic blood pressure, and respiratory rate each had one unavailable observation, while Mallampati class had three. These entries were excluded from the corresponding descriptive summaries without imputation. All other variables reported in Supplementary Table S1 had complete data for the 109 participants.

Table S2: Missing-data summary after verification.

Variable	Available n	Missing n	Reason / handling
Systolic blood pressure	108	1	Unrecoverable capture error; treated as missing
Diastolic blood pressure	108	1	Unrecoverable capture error; treated as missing
Respiratory rate	108	1	Unrecoverable capture error; treated as missing
Mallampati class	106	3	Database code 0 denotes unavailable data

Additional note. All other variables in Supplementary Table S1 had complete data for the 109 participants. No imputation was performed for the missing values listed above.

Availability of segmented instances

Supplementary Table S3 lists the 15 participants who contributed fewer than the 30 expected segmented instances. For each participant, the table reports the study identifier, AHI, binary screening class, and numbers of valid and discarded instances. Rows are ordered by decreasing number of discarded instances. The remaining 94 participants contributed all 30 expected instances. Overall, 117 of the 3270 expected instances were discarded because some frames were too short for reliable acoustic feature extraction, leaving 3153 valid instances. All 109 participants remained represented in the segmented dataset.

Table S3: Participant-level summary of valid and discarded segmented instances.

Participant	AHI	Class	Valid instances	Discarded
46	8.7	AHI < 10	7	23
115	64.5	AHI ≥ 10	12	18
101	52.5	AHI ≥ 10	16	14
82	37.1	AHI ≥ 10	18	12
144	0.7	AHI < 10	18	12
67	28.3	AHI ≥ 10	22	8
62	61.7	AHI ≥ 10	24	6
70	4.7	AHI < 10	25	5
98	0.0	AHI < 10	25	5
110	1.6	AHI < 10	26	4
73	22.3	AHI ≥ 10	26	4
72	5.8	AHI < 10	27	3
29	18.4	AHI ≥ 10	29	1
99	1.4	AHI < 10	29	1
69	77.2	AHI ≥ 10	29	1

Note. Ninety-four participants contributed all 30 expected segmented instances. Fifteen participants had one or more discarded instances because some frames were too short for reliable acoustic feature extraction.

Hyperparameter search settings

Supplementary Table S4 summarizes the parameters included in the inner cross-validation searches for the nine classification and eight regression algorithms evaluated in this study. Parameters are listed separately by task and model. The table covers the algorithms used with the unsegmented and segmented representations; fixed settings and parameters without an effect under the selected solver are identified in the notes.

Table S4: Parameters included in the inner cross-validation searches for classification and regression algorithms.

Task	Model	Parameters included in the search
Classification	SVC	C, kernel, gamma, class_weight
Classification	GNB	var_smoothing
Classification	QDA	reg_param
Classification	KNN	n_neighbors, weights, metric
Classification	DT	criterion, max_depth, min_samples_split, class_weight
Classification	RF	n_estimators, criterion, max_depth, min_samples_split, class_weight
Classification	MLPC	hidden_layer_sizes, activation, solver, alpha, batch_size, learning_rate, max_iter
Classification	LRC	C, solver, class_weight
Classification	XGBC	n_estimators, max_depth, learning_rate, subsample, colsample_bytree, scale_pos_weight
Regression	SVR	C, kernel, gamma
Regression	DT	criterion, splitter, max_depth, min_samples_split, min_samples_leaf
Regression	SGD	loss, max_iter, tol
Regression	Lasso	alpha
Regression	Ridge	alpha
Regression	RF	n_estimators, max_depth, min_samples_split, min_samples_leaf
Regression	MLPR	hidden_layer_sizes, activation, solver, alpha, max_iter
Regression	XGBR	n_estimators, max_depth, learning_rate, subsample, colsample_bytree

Notes. Hyperparameter selection was performed within the four-fold inner cross-validation loop, using only the training portion of each outer fold. RandomizedSearchCV evaluated up to 20 candidate configurations per search; fewer configurations were evaluated when the predefined search space contained fewer than 20 combinations. Classification configurations were selected by maximizing AUC-ROC, whereas regression configurations were selected by minimizing MAE.

Class-imbalance handling was included through class_weight for SVC, DT, RF, and LRC, and through scale_pos_weight for XGBC.

For MLPC and MLPR, solver was included in the search specification but had only one candidate value, adam; therefore, alternative solvers were not compared. For MLPC, learning_rate was also included in the search specification, but this parameter controls the learning-rate schedule only when solver=sgd and had no effect with solver=adam.

SVC = support vector classifier; GNB = Gaussian naive Bayes; QDA = quadratic discriminant analysis; KNN = k-nearest neighbors; DT = decision tree; RF = random forest; MLPC = multilayer perceptron

classifier; LRC = logistic regression classifier; XGBC = extreme gradient boosting classifier; SVR = support vector regression; SGD = stochastic gradient descent regressor; MLPR = multilayer perceptron regressor; XGBR = extreme gradient boosting regressor; AUC-ROC = area under the receiver operating characteristic curve; MAE = mean absolute error.

Regression MAE with unsegmented features

Supplementary Table S5 presents the mean absolute error (MAE) of the eight regression algorithms evaluated across the seven unsegmented feature configurations in the primary cohort of 109 participants. Rows correspond to regression algorithms, and columns correspond to craniofacial, acoustic, and combined feature sets. Results are summarized as mean ± standard deviation across the 10 complete outer cross-validation repetitions. The lowest mean MAE within each feature set is highlighted in bold.

Table S5: Mean absolute error of regression algorithms using unsegmented feature sets in the 109-participant cohort.

Model	CF	V	P	V+P	CF+V	CF+P	CF+V+P
SVR	**14.537 ± 0.497**	**16.328 ± 0.390**	**16.085 ± 0.301**	**16.040 ± 0.254**	15.902 ± 0.181	15.461 ± 0.423	15.992 ± 0.248
DT	17.426 ± 1.421	18.815 ± 1.586	19.522 ± 0.700	20.058 ± 1.754	19.262 ± 0.806	18.884 ± 1.223	19.006 ± 0.948
SGD	15.296 ± 0.404	17.524 ± 0.624	18.338 ± 1.187	18.158 ± 0.620	16.297 ± 0.551	**15.327 ± 0.259**	17.110 ± 0.595
Lasso	15.532 ± 0.324	16.502 ± 0.408	16.993 ± 1.197	16.714 ± 0.431	15.853 ± 0.319	15.858 ± 0.279	16.183 ± 0.335
Ridge	15.739 ± 0.363	19.404 ± 1.305	18.588 ± 0.740	23.216 ± 1.326	19.923 ± 0.983	18.692 ± 0.825	23.264 ± 0.987
RF	15.342 ± 0.478	16.986 ± 0.533	16.680 ± 0.357	16.743 ± 0.442	**15.667 ± 0.419**	15.463 ± 0.353	**15.700 ± 0.404**
MLPR	14.903 ± 0.514	18.161 ± 0.784	18.557 ± 0.890	19.088 ± 0.591	17.247 ± 0.650	17.996 ± 0.561	18.634 ± 0.794
XGBR	15.606 ± 0.373	16.837 ± 0.668	16.726 ± 0.448	16.885 ± 0.588	16.074 ± 0.545	15.420 ± 0.420	16.140 ± 0.415

Notes. MAE is expressed in events/h; lower values indicate better predictive performance. Within each outer cross-validation repetition, MAE was calculated from the participant-level out-of-fold predictions pooled across the five outer test folds. The reported standard deviation describes variability across the 10 complete repetitions and is not a confidence interval. Bold identifies the lowest mean MAE within each feature set and does not imply statistically significant differences between algorithms.

CF = craniofacial measurements; V = sustained-vowel acoustic features; P = continuous-speech acoustic features; the plus sign denotes combined feature sets. SVR = support vector regression; DT = decision tree; SGD = stochastic gradient descent regressor; RF = random forest; MLPR = multilayer perceptron regressor; XGBR = extreme gradient boosting regressor.

Regression RMSE with unsegmented features

Supplementary Table S6 presents the root mean squared error (RMSE) of the eight regression algorithms evaluated across the seven unsegmented feature configurations in the primary cohort of 109 participants. Rows correspond to regression algorithms, and columns correspond to craniofacial, acoustic, and combined feature sets. Results are summarized as mean ± standard deviation across the 10 complete outer cross-validation repetitions. The lowest mean RMSE within each feature set is highlighted in bold.

Table S6: Root mean squared error of regression algorithms using unsegmented feature sets in the 109-participant cohort.

Model	CF	V	P	V+P	CF+V	CF+P	CF+V+P
SVR	19.904 ± 0.402	21.504 ± 0.410	21.225 ± 0.400	21.268 ± 0.340	21.219 ± 0.281	20.550 ± 0.369	21.125 ± 0.259
DT	22.910 ± 1.541	24.165 ± 1.742	25.431 ± 1.074	25.883 ± 2.227	25.253 ± 1.134	24.843 ± 1.784	25.764 ± 1.238
SGD	20.801 ± 0.558	23.999 ± 0.639	24.115 ± 1.994	24.501 ± 0.679	22.509 ± 0.461	20.748 ± 0.240	23.091 ± 0.491
Lasso	20.152 ± 0.311	**20.870 ± 0.460**	21.871 ± 1.822	20.956 ± 0.532	20.270 ± 0.358	20.126 ± 0.463	20.384 ± 0.455
Ridge	20.205 ± 0.435	25.050 ± 1.499	24.587 ± 1.084	30.848 ± 2.307	25.036 ± 1.269	23.555 ± 1.211	30.824 ± 2.371
RF	**19.867 ± 0.586**	21.101 ± 0.629	**20.760 ± 0.552**	**20.810 ± 0.597**	**19.977 ± 0.497**	**19.673 ± 0.572**	**19.954 ± 0.567**
MLPR	20.580 ± 0.586	23.537 ± 1.166	23.980 ± 1.167	24.748 ± 0.974	23.130 ± 0.909	23.879 ± 1.034	24.421 ± 1.005
XGBR	20.191 ± 0.448	21.285 ± 0.924	21.059 ± 0.765	21.251 ± 0.680	20.733 ± 0.676	19.861 ± 0.716	20.604 ± 0.540

Notes. RMSE is expressed in events/h; lower values indicate better predictive performance. Within each outer cross-validation repetition, RMSE was calculated from the participant-level out-of-fold predictions pooled across the five outer test folds. The reported standard deviation describes variability across the 10 complete repetitions and is not a confidence interval. Bold identifies the lowest mean RMSE within each feature set and does not imply statistically significant differences between algorithms. Hyperparameter selection used MAE rather than RMSE.

CF = craniofacial measurements; V = sustained-vowel acoustic features; P = continuous-speech acoustic features; the plus sign denotes combined feature sets. SVR = support vector regression; DT = decision tree; SGD = stochastic gradient descent regressor; RF = random forest; MLPR = multilayer perceptron regressor; XGBR = extreme gradient boosting regressor.

Regression MAE with segmented features

Supplementary Table S7 presents the mean absolute error (MAE) of the eight regression algorithms evaluated across the seven segmented feature configurations in the primary cohort of 109 participants. Rows correspond to regression algorithms, and columns correspond to craniofacial, acoustic, and combined feature sets. Performance was evaluated at the participant level after averaging predictions from segmented instances. Results are summarized as mean ± standard deviation across the 10 complete outer cross-validation repetitions. The lowest mean MAE within each feature set is highlighted in bold.

Table S7: Mean absolute error of regression algorithms using segmented feature sets in the 109-participant cohort.

Model	CF	V	P	V+P	CF+V	CF+P	CF+V+P
SVR	**14.713 ± 0.485**	16.101 ± 0.245	15.940 ± 0.136	16.143 ± 0.207	15.353 ± 0.529	14.829 ± 0.485	15.440 ± 0.516
DT	17.690 ± 1.229	16.483 ± 0.477	14.567 ± 0.372	15.939 ± 0.402	18.123 ± 1.731	17.875 ± 1.417	17.514 ± 1.143
SGD	15.188 ± 0.327	16.790 ± 0.562	15.687 ± 0.280	16.360 ± 0.576	15.669 ± 0.467	**14.761 ± 0.334**	15.129 ± 0.429
Lasso	15.403 ± 0.312	**15.934 ± 0.202**	15.772 ± 0.151	15.818 ± 0.160	15.429 ± 0.470	15.227 ± 0.266	15.341 ± 0.508
Ridge	16.034 ± 0.375	16.451 ± 0.371	15.765 ± 0.161	16.328 ± 0.382	17.123 ± 0.561	16.054 ± 0.320	17.044 ± 0.496
RF	17.039 ± 1.052	16.470 ± 0.492	**14.242 ± 0.208**	16.017 ± 0.461	16.096 ± 0.794	16.763 ± 0.983	16.086 ± 0.723
MLPR	18.815 ± 1.914	17.236 ± 0.515	15.848 ± 0.266	17.109 ± 0.670	16.265 ± 0.931	17.947 ± 1.742	16.498 ± 0.565
XGBR	15.725 ± 0.602	16.082 ± 0.391	14.593 ± 0.174	**15.787 ± 0.353**	**15.150 ± 0.704**	15.082 ± 0.422	**15.116 ± 0.729**

Notes. MAE is expressed in events/h; lower values indicate better predictive performance. Within each outer test fold, predictions from segmented instances belonging to the same participant were averaged before evaluation. Within each outer cross-validation repetition, MAE was calculated from the participant-level out-of-fold predictions pooled across the five outer test folds. The reported standard deviation describes variability across the 10 complete repetitions and is not a confidence interval. Bold identifies the lowest mean MAE within each feature set and does not imply statistically significant differences between algorithms.

CF = craniofacial measurements; V = sustained-vowel acoustic features; P = continuous-speech acoustic features; the plus sign denotes combined feature sets. SVR = support vector regression; DT = decision tree; SGD = stochastic gradient descent regressor; RF = random forest; MLPR = multilayer perceptron regressor; XGBR = extreme gradient boosting regressor.

Regression RMSE with segmented features

Supplementary Table S8 presents the root mean squared error (RMSE) of the eight regression algorithms evaluated across the seven segmented feature configurations in the primary cohort of 109 participants. Rows correspond to regression algorithms, and columns correspond to craniofacial, acoustic, and combined feature sets. Performance was evaluated at the participant level after averaging predictions from segmented instances. Results are summarized as mean ± standard deviation across the 10 complete outer cross-validation repetitions. The lowest mean RMSE within each feature set is highlighted in bold.

Table S8: Root mean squared error of regression algorithms using segmented feature sets in the 109-participant cohort.

Model	CF	V	P	V+P	CF+V	CF+P	CF+V+P
SVR	**20.029 ± 0.494**	21.330 ± 0.291	21.096 ± 0.196	21.383 ± 0.305	20.489 ± 0.530	20.197 ± 0.448	20.599 ± 0.530
DT	23.874 ± 1.391	20.899 ± 0.689	18.486 ± 0.624	20.101 ± 0.571	24.201 ± 2.387	23.761 ± 1.840	23.280 ± 1.420
SGD	20.765 ± 0.459	21.603 ± 0.718	20.795 ± 0.282	21.356 ± 0.665	21.103 ± 0.553	20.309 ± 0.227	20.737 ± 0.349
Lasso	20.054 ± 0.335	20.517 ± 0.361	20.436 ± 0.156	20.399 ± 0.316	19.990 ± 0.475	**19.782 ± 0.226**	19.868 ± 0.505
Ridge	20.425 ± 0.476	20.872 ± 0.497	20.228 ± 0.186	20.755 ± 0.481	21.286 ± 0.557	20.363 ± 0.350	21.291 ± 0.448
RF	22.449 ± 1.515	20.524 ± 0.749	**17.920 ± 0.246**	20.048 ± 0.666	21.084 ± 0.946	22.052 ± 1.497	20.997 ± 0.897
MLPR	25.541 ± 3.782	21.802 ± 0.806	20.145 ± 0.295	21.790 ± 0.917	21.016 ± 1.336	24.509 ± 4.236	21.331 ± 1.116
XGBR	20.540 ± 0.967	**20.028 ± 0.626**	18.314 ± 0.283	**19.689 ± 0.440**	**19.781 ± 1.015**	19.795 ± 0.745	**19.679 ± 1.138**

Notes. RMSE is expressed in events/h; lower values indicate better predictive performance. Within each outer test fold, predictions from segmented instances belonging to the same participant were averaged before evaluation. Within each outer cross-validation repetition, RMSE was calculated from the participant-level out-of-fold predictions pooled across the five outer test folds. The reported standard deviation describes variability across the 10 complete repetitions and is not a confidence interval. Bold identifies the lowest mean RMSE within each feature set and does not imply statistically significant differences between algorithms. Hyperparameter selection used MAE rather than RMSE.

CF = craniofacial measurements; V = sustained-vowel acoustic features; P = continuous-speech acoustic features; the plus sign denotes combined feature sets. SVR = support vector regression; DT = decision tree; SGD = stochastic gradient descent regressor; RF = random forest; MLPR = multilayer perceptron regressor; XGBR = extreme gradient boosting regressor.

Classification AUC-ROC with unsegmented features

Supplementary Table S9 presents the area under the receiver operating characteristic curve (AUC-ROC) for the classification algorithms evaluated across the seven unsegmented feature configurations in the primary cohort of 109 participants. Rows correspond to classification algorithms, and columns correspond to craniofacial, acoustic, and combined feature sets. Results are summarized as mean ± standard deviation across the 10 complete outer cross-validation repetitions. The highest mean AUC-ROC within each feature set is highlighted in bold. Dashes identify configurations that were not evaluated.

Table S9: AUC-ROC of classification algorithms using unsegmented feature sets in the 109-participant cohort.

Model	CF	V	P	V+P	CF+V	CF+P	CF+V+P
SVC	0.731 ± 0.030	0.516 ± 0.034	0.490 ± 0.038	0.523 ± 0.038	0.583 ± 0.043	0.636 ± 0.040	0.590 ± 0.036
GNB	0.722 ± 0.029	0.515 ± 0.036	0.480 ± 0.047	0.493 ± 0.036	0.595 ± 0.034	0.601 ± 0.032	0.555 ± 0.037
QDA	0.655 ± 0.045	—	—	—	—	—	—
KNN	0.676 ± 0.033	0.558 ± 0.037	0.527 ± 0.036	0.545 ± 0.041	0.611 ± 0.040	0.566 ± 0.026	0.550 ± 0.039
DT	0.658 ± 0.033	0.490 ± 0.057	0.484 ± 0.049	0.482 ± 0.042	0.610 ± 0.029	0.577 ± 0.048	0.580 ± 0.047
RF	0.752 ± 0.040	0.549 ± 0.039	**0.550 ± 0.028**	0.532 ± 0.033	**0.704 ± 0.021**	**0.685 ± 0.030**	**0.678 ± 0.026**
MLPC	**0.766 ± 0.036**	**0.571 ± 0.040**	0.515 ± 0.032	**0.578 ± 0.036**	0.695 ± 0.024	0.656 ± 0.034	0.646 ± 0.037
LRC	0.735 ± 0.028	0.528 ± 0.047	0.512 ± 0.053	0.505 ± 0.044	0.640 ± 0.046	0.656 ± 0.032	0.618 ± 0.048
XGBC	0.716 ± 0.047	0.506 ± 0.039	0.533 ± 0.027	0.516 ± 0.040	0.664 ± 0.037	0.661 ± 0.037	0.632 ± 0.036

Notes. Higher AUC-ROC values indicate better discrimination. Within each outer cross-validation repetition, AUC-ROC was calculated from the participant-level out-of-fold scores pooled across the five outer test folds. The reported standard deviation describes variability across the 10 complete repetitions and is not a confidence interval. Bold identifies the highest mean AUC-ROC within each feature set and does not imply statistically significant differences between algorithms.

QDA was evaluated only for CF in the unsegmented representation. Configurations containing acoustic features were excluded from QDA evaluation because their dimensionality relative to the available class-specific training samples limited reliable estimation of class-specific covariance matrices. Dashes denote configurations that were not evaluated.

CF = craniofacial measurements; V = sustained-vowel acoustic features; P = continuous-speech acoustic features; the plus sign denotes combined feature sets. SVC = support vector classifier; GNB = Gaussian naive Bayes; QDA = quadratic discriminant analysis; KNN = k-nearest neighbors; DT = decision tree; RF = random forest; MLPC = multilayer perceptron classifier; LRC = logistic regression classifier; XGBC = extreme gradient boosting classifier.

Classification AUC-ROC with segmented features

Supplementary Table S10 presents the area under the receiver operating characteristic curve (AUC-ROC) for the nine classification algorithms evaluated across the seven segmented feature configurations in the primary cohort of 109 participants. Rows correspond to classification algorithms, and columns correspond to craniofacial, acoustic, and combined feature sets. Performance was evaluated at the participant level after averaging scores from segmented instances. Results are summarized as mean ± standard deviation across the 10 complete outer cross-validation repetitions. The highest mean AUC-ROC within each feature set is highlighted in bold.

Table S10: AUC-ROC of classification algorithms using segmented feature sets in the 109-participant cohort.

Model	CF	V	P	V+P	CF+V	CF+P	CF+V+P
SVC	0.725 ± 0.051	0.452 ± 0.054	0.550 ± 0.035	0.458 ± 0.042	0.727 ± 0.046	0.732 ± 0.032	0.747 ± 0.040
GNB	0.723 ± 0.033	0.442 ± 0.031	0.494 ± 0.038	0.444 ± 0.030	0.558 ± 0.059	0.672 ± 0.031	0.557 ± 0.054
QDA	0.668 ± 0.028	0.460 ± 0.042	0.507 ± 0.036	0.454 ± 0.057	0.612 ± 0.046	0.642 ± 0.019	0.586 ± 0.046
KNN	0.642 ± 0.027	0.495 ± 0.020	0.483 ± 0.036	0.412 ± 0.038	0.597 ± 0.030	0.648 ± 0.018	0.556 ± 0.051
DT	0.657 ± 0.048	**0.521 ± 0.050**	0.560 ± 0.042	0.507 ± 0.054	0.674 ± 0.044	0.669 ± 0.037	0.659 ± 0.050
RF	**0.753 ± 0.022**	0.489 ± 0.035	0.549 ± 0.021	0.506 ± 0.037	0.749 ± 0.026	**0.756 ± 0.030**	0.748 ± 0.022
MLPC	0.749 ± 0.044	0.466 ± 0.024	0.545 ± 0.035	0.488 ± 0.043	**0.751 ± 0.029**	0.751 ± 0.026	0.730 ± 0.029
LRC	0.743 ± 0.036	0.512 ± 0.020	0.554 ± 0.021	**0.510 ± 0.030**	0.750 ± 0.038	0.747 ± 0.022	**0.749 ± 0.033**
XGBC	0.693 ± 0.041	0.495 ± 0.038	**0.568 ± 0.018**	0.485 ± 0.048	0.719 ± 0.034	0.703 ± 0.038	0.714 ± 0.037

Notes. Higher AUC-ROC values indicate better discrimination. Within each outer test fold, scores from segmented instances belonging to the same participant were averaged before evaluation. Within each outer cross-validation repetition, AUC-ROC was calculated from the participant-level out-of-fold scores pooled across the five outer test folds. The reported standard deviation describes variability across the 10 complete repetitions and is not a confidence interval. Bold identifies the highest mean AUC-ROC within each feature set and does not imply statistically significant differences between algorithms.

CF = craniofacial measurements; V = sustained-vowel acoustic features; P = continuous-speech acoustic features; the plus sign denotes combined feature sets. SVC = support vector classifier; GNB = Gaussian naive Bayes; QDA = quadratic discriminant analysis; KNN = k-nearest neighbors; DT = decision tree; RF = random forest; MLPC = multilayer perceptron classifier; LRC = logistic regression classifier; XGBC = extreme gradient boosting classifier.
